# Bacterial community structure alterations within the colorectal cancer gut microbiome

**DOI:** 10.1186/s12866-021-02153-x

**Published:** 2021-03-31

**Authors:** Mark Loftus, Sayf Al-Deen Hassouneh, Shibu Yooseph

**Affiliations:** 1 Burnett School of Biomedical Sciences, Genomics and Bioinformatics Cluster, University of Central Florida, Orlando, 32816 FL USA; 2 Department of Computer Science, Genomics and Bioinformatics Cluster, University of Central Florida, Orlando, FL 32816 USA

**Keywords:** Colorectal, Cancer, Microbiome, Metagenomics, Networks, Oral, Pathogens, Associations

## Abstract

**Background:**

Colorectal cancer is a leading cause of cancer-related deaths worldwide. The human gut microbiome has become an active area of research for understanding the initiation, progression, and treatment of colorectal cancer. Despite multiple studies having found significant alterations in the carriage of specific bacteria within the gut microbiome of colorectal cancer patients, no single bacterium has been unequivocally connected to all cases. Whether alterations in species carriages are the cause or outcome of cancer formation is still unclear, but what is clear is that focus should be placed on understanding changes to the bacterial community structure within the cancer-associated gut microbiome.

**Results:**

By applying a novel set of analyses on 252 previously published whole-genome shotgun sequenced fecal samples from healthy and late-stage colorectal cancer subjects, we identify taxonomic, functional, and structural changes within the cancer-associated human gut microbiome. Bacterial association networks constructed from these data exhibited widespread differences in the underlying bacterial community structure between healthy and colorectal cancer associated gut microbiomes. Within the cancer-associated ecosystem, bacterial species were found to form associations with other species that are taxonomically and functionally dissimilar to themselves, as well as form modules functionally geared towards potential changes in the tumor-associated ecosystem. Bacterial community profiling of these samples revealed a significant increase in species diversity within the cancer-associated gut microbiome, and an elevated relative abundance of species classified as originating from the oral microbiome including, but not limited to, *Fusobacterium nucleatum*, *Peptostreptococcus stomatis*, *Gemella morbillorum*, and *Parvimonas micra*. Differential abundance analyses of community functional capabilities revealed an elevation in functions linked to virulence factors and peptide degradation, and a reduction in functions involved in amino-acid biosynthesis within the colorectal cancer gut microbiome.

**Conclusions:**

We utilize whole-genome shotgun sequenced fecal samples provided from a large cohort of late-stage colorectal cancer and healthy subjects to identify a number of potentially important taxonomic, functional, and structural alterations occurring within the colorectal cancer associated gut microbiome. Our analyses indicate that the cancer-associated ecosystem influences bacterial partner selection in the native microbiota, and we highlight specific oral bacteria and their associations as potentially relevant towards aiding tumor progression.

**Supplementary Information:**

The online version contains supplementary material available at 10.1186/s12866-021-02153-x.

## Background

The human gastrointestinal tract harbors a highly diverse community of bacterial cells thought to be in comparable abundance to those of its human host making it the largest and most complex community of bacteria found associating with the human body [1]. These bacteria are typically regarded as commensal, or symbiotic, in that they generally cause no harm and provide fundamental services for their host’s nutrition and continued health. The most important of these services include the creation of metabolic by-products (short chain fatty acids, hormones, vitamins, etc.), aiding in proper intestinal tissue and immune system development and regulation, and protecting the gut from colonization by pathogenic organisms [2, 3]. Many diseases have been associated with the disruption of the gut microbiome’s bacterial community, one of which is colorectal cancer (CRC) [4–7].

CRC is one of the leading causes of cancer-related deaths worldwide [8] and is characterized by the uncontrolled growth of epithelial cells within the colon or rectum. The transformation of epithelial cells from noncancerous to cancerous growth commonly begins with the formation of a polyp, which over a 10-to-20-year period may or may not progress to become an invasive cancer [9]. CRC initiation is understood as being the result of a combination of both genetic and environmental factors (diet, smoking, alcohol, etc.) [10–12], although the majority (around 75%) of CRC cases are spontaneous, with genetic risk factors being attributed to less than 10% of cases [13, 14]. Recently, there has been a surge in evidence supporting the hypothesis that the human gut microbiome plays a prominent role in relation to cancer initiation, progression, and in the efficacy of its treatment [7, 15–20]. One of the leading hypotheses is the “driver-passenger” model [17], which postulates that a “driver” bacterium such as *Fusobacterium nucleatum*, *Bacteroides fragilis*, or *Escherichia coli* promotes genomic instability (damage) to the DNA of epithelial cells, potentially through some virulence factor, which leads to cellular mutation and eventually tumor formation. Following tumor formation, the changes in micro-environmental conditions around the tumor mass (tumor microenvironment; TME) would optimize the growth of “passenger” microbes who are better suited to this niche facilitating their colonization, and eventual out-competing of the “driver” species as well as the native microbiota leading to a depletion in protective commensal species. These “passenger” microbes could either be pathogens that exist normally in the healthy gut microbiome in low abundance, or simply commensal bacteria that have acquired pathogenic characteristics due to the alteration in the local intestinal ecology. As of now, there is no consistent cancer-associated community profile that has been observed leaving researchers with limited understanding of the full extent the microbiota plays in CRC. Nevertheless, the modulation of the bacterial community within the cancer-associated gut microbiome is the next logical step in possible CRC treatment and prevention strategies.

To one day utilize the bacterial community toward these purposes, it is important to know more than which species are present or absent in the community during disease. We also need to understand how the associations between bacterial species have been affected. These associations are shaped by both direct and indirect interactions taking place in the community (e.g., cooperation or competition), and are important as they are the bedrock upon which the community services, as well as the structure and function, are founded on [21, 22]. In this study, we represent these associations using a weighted graph (network) in which a node denotes a bacterial species and a weighted edge between two nodes represents the strength of the association between the corresponding species. By using this framework, we can model the positive and negative associations between species, thereby shedding light on how cooperation and competition shape the structure of the bacterial community. Bacterial association networks are constructed from sample-taxa count matrices. A sample-taxa count matrix is commonly generated by sequencing the collected biological samples and determining the taxa (species) counts in each sample. However, DNA sequencing does not provide the absolute counts of these taxa within a sample, and instead provides only their relative abundances (i.e., compositional data) [23]. Due to this aspect, inferring associations between species is challenging, and using measures like correlation can produce misleading results when applied directly to compositional data [24]. With this limitation in mind, we applied a Gaussian Graphical Model (GGM) framework on Centered Log-Ratio (CLR) transformed sequence count data to model the conditional dependencies between species to construct association networks [25]. Prior studies that investigated the associations between bacteria within the CRC-associated gut microbiome have either not dealt appropriately with compositional data (for instance, application of correlation directly to untransformed data), or have utilized low taxonomic resolution data (16S rRNA data) which should be used cautiously to assign taxonomic classifications beneath genus-level [5, 26–30]. For the analysis presented here, we utilize 252 whole-genome shotgun (WGS) sequenced fecal samples provided by healthy and late-stage (stage III and IV) CRC subjects from a previously published study [31] to investigate bacterial associations at the species level [32]. The authors of that study originally performed metagenomic and metabolomic analyses to assess any taxonomic and functional differences of the gut microbiota, and metabolites, as well as find diagnostic markers for CRC. For their analyses, these researchers only focused on finding alterations of the microbiota pertaining to species currently known to be culturable and constructed bacterial association networks using correlation (Spearman’s) at the genus-level. Our analysis framework and goals are different. For our study, we used a comprehensive collection of nearly eleven thousand bacterial strain reference genomes from NCBI’s RefSeq database to calculate the genome relative abundance of bacterial species in each sample using an Expectation-Maximization (EM) algorithm. Subsequently, species were selected based on their prevalence, relative abundance, and feature importance, and were used to construct bacterial association networks using the graphical lasso (glasso) approach [33]. These networks were then analyzed to assess the differences in bacterial community structure between the healthy and late-stage CRC-associated gut microbiome. Taxonomic and functional analysis was performed to highlight differences in gut microbiome bacterial community functional capabilities and species carriages. Our results not only identify both individual and groups (modules) of species potentially capable of aiding tumor progression, but also shows how the bacterial community structure has dramatically altered in response to potential ecological changes occurring within the CRC-associated gut microbiome.

## Results

### Bacterial community taxonomic profiling

**Fig. 1:**
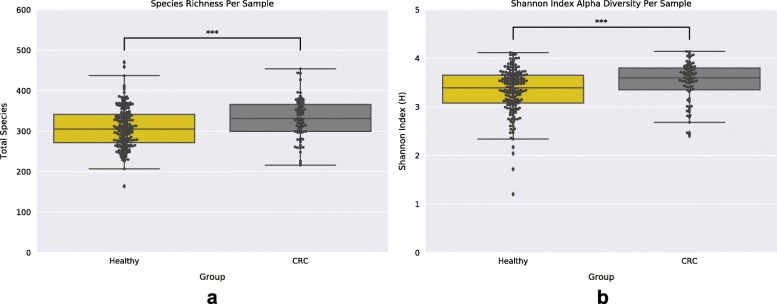
Species Diversity Within Healthy and CRC Samples. **a**. Boxplot of sample species richness (total species) showing significantly greater species richness within the CRC sample group. **b**. Boxplot of sample Shannon diversity shows significantly greater species diversity within CRC sample group. Black dots represent individual samples and stars (***) denote statistical significance (MWU pvalue < 0.001)

**Fig. 2:**
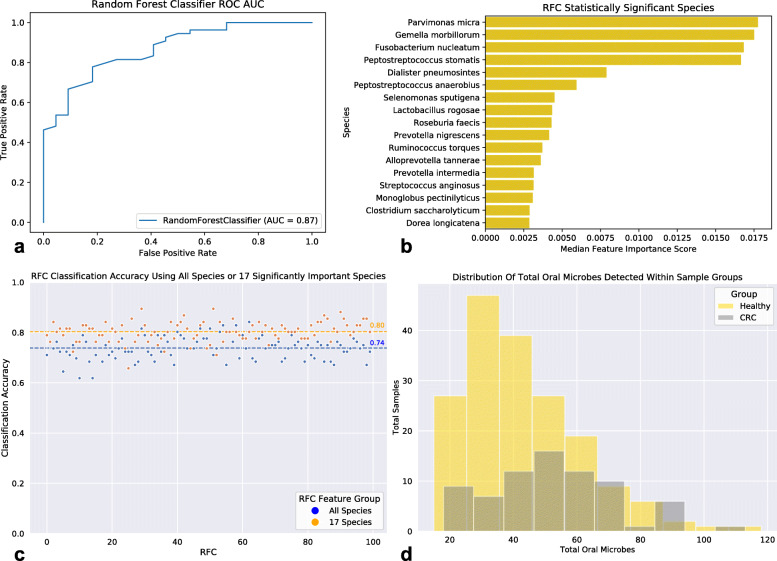
Healthy and CRC Taxonomic Profiling. **a**. Random Forest Classifier (RFC) ROC showing an AUC = 0.87. **b**. Seventeen statistically significant (MWU-FDR qvalue< 0.05) ‘important’ species from 100 RFCs compared to a random feature. **c**. Classification accuracy of 100 RFCs using either all species or the 17 significantly ‘important’ species. **d**. Distribution of total oral microbes within Healthy and CRC sample groups. A significantly (MWU pvalue< 0.05) greater total population of oral microbes were found in the CRC-associated gut microbiome. Bacterial species were classified as oral microbes by using the expanded Human Oral Microbe Database (eHOMD)

Following sample pre-processing (see methods), we computed the relative abundance of species within each sample using an EM-based method in order to construct a sample-taxa matrix (see methods). This sample-taxa matrix was then used to investigate the bacterial community diversity in the two sample groups (Healthy and late-stage CRC) by measuring the bacterial richness and Shannon index of each sample. Samples originating from the CRC group exhibited significantly greater diversity, both richness and Shannon index, (Mann-Whitney U test: MWU); Richness: MWU pvalue = 0.0005 and Shannon Index: MWU pvalue = 0.0009) compared to those of the Healthy group (Fig. 1a,b). Considering that measures of species diversity differ in their sensitivity to species evenness and richness [34], we additionally applied the Simpson index of diversity to compute species diversity within sample groups (see supplemental file). These results were congruent with our previous analyses showing a statistically significant (MWU: pvalue = 0.0238) higher species diversity in CRC samples compared to that found in Healthy samples (Supplemental 1). We next assessed the differences in bacterial community taxonomic profiles between the healthy and late-stage CRC-associated gut microbiomes. Prior to performing further analyses we applied a CLR-transformation to our sample-taxa matrix (see methods). Taxonomic profile-based sample ordination was carried out using Principal Components Analysis (PCA). The first two principal components explain only a small fraction of the total variance (PC1: 7.98%, PC2: 5.61%) (Supplemental 2), and the linear transformation based on PCA did not show evidence for separation of Healthy samples from CRC samples. However, we were able to distinguish between the two sample groups using a Random Forest Classifier (RFC) (AUC = 0.87) (Fig. 2a). While RFCs rank features (species) based on their importance, these default measures of importance are known to be biased and lead to the return of suboptimal predictor features [35]. To obtain statistical significance for species importances provided by the RFC we applied a technique where we included a “random” feature into our feature set (see methods). By using an ensemble of 100 RFCs we uncovered 17 bacterial species that were statistically (MWU and False Discovery Rate Multiple Testing Correction; MWU-FDR: qvalue< 0.05) more ‘important’ (deemed significantly ‘important’) than the random feature for distinguishing groups (Fig. 2b). We found that the accuracy classification score of 100 RFCs trained on the 17 significantly ‘important’ species was on average greater than that of the 100 RFCs trained on all species (All Species Mean Accuracy: 74%; 17 significantly ‘important’ Species Mean Accuracy: 80%) (Fig. 2c). We next performed species differential abundance analysis (see methods) which revealed 174 species significantly (MWU-FDR qvalue< 0.05) reduced in relative abundance, and 10 species significantly elevated in relative abundance within the CRC-associated gut microbiome compared to the Healthy gut microbiome. These 174 bacterial species are from a diverse background of 84 genera, although the largest fraction of species were from the genera *Enterobacter* (6.8%), *Klebsiella* (6.3%), *Streptococcus* (5.2%), *Lactobacillus* (5.1%), *Citrobacter* (4.6%), *Bifidobacterium* (4%), *Bacteroides* (3.4%), and *Clostridium* (3.4%) (Supplemental 3). The 10 species significantly elevated in relative abundance within CRC were: *Parvimonas micra* (qvalue = 3.09e-09), *Peptostreptococcus stomatis* (qvalue = 4.51e-08), *Gemella morbillorum* (qvalue = 4.55e-08), *Fusobacterium nucleatum* (qvalue = 1.08e-06), *Streptococcus anginosus* (qvalue = 1.13e-03), *Dialister pneumosintes* (qvalue = 1.37e-03), *Peptostreptococcus anaerobius* (qvalue = 4.74e-03), *Streptococcus sp. KCOM 2412* (*Streptococcus periodonticum*) (qvalue = 7.18e-03), *Ruminococcus torques* (qvalue = 1.55e-02), and *Filifactor alocis* (qvalue = 2.85e-02) (Supplemental 4a-c). Interestingly, many of the species that were deemed both significantly ‘important’ and elevated in relative abundance within CRC are also found in the oral microbiome and noted to be associated with oral diseases (periodontitis, periapical lesions, root canal infections, oral cancers, etc.) which have been associated with increased risks of CRC [36–45]. Subsequently, we utilized the expanded Human Oral Microbe Database (eHOMD) [46] to classify all oral species within our samples and found a significant increase in the total oral microbe population richness existing within the CRC-associated gut microbiome in comparison to that of the Healthy group (MWU: pvalue = 6.51e-05) (Fig. 2d).

### Bacterial community functional profiling

To analyze the differences in community functional capabilities between the Healthy and CRC gut microbiomes we measured the relative abundance of protein families (TIGRFAMs [47]) and protein domains (Pfams [48]) within our WGS samples creating a sample-function matrix (see methods). A CLR-transformation was applied to this matrix and then PCA was performed. PCA showed evidence of inter-group clustering of samples (Healthy and CRC) and ultimately only explained a moderate variance (PC1: 27.19%, PC2: 4.33%) (Supplemental 5). Differential abundance analysis was performed using the CLR-transformed sample-function matrix which showed 12 Pfams (7 elevated and 5 reduced in CRC compared to Healthy) and two TIGRFAMs (1 elevated and 1 reduced in CRC compared to Healthy) to be statistically significantly (MWU-FDR: qvalue< 0.05) different in their relative abundance (Supplemental Table 1). Pfams that were significantly elevated within the CRC gut microbiome were linked to bacterial invasins and adhesins (ex: FadA), while those that were significantly reduced were tied to antibiotic resistance, bacteriophage maturation, and threonine biosynthesis. The single TIGRFAM significantly elevated in CRC was linked to proline iminopeptidase, while the only TIGRFAM significantly reduced was again linked to threonine biosynthesis.

### Bacterial association networks

**Fig. 3:**
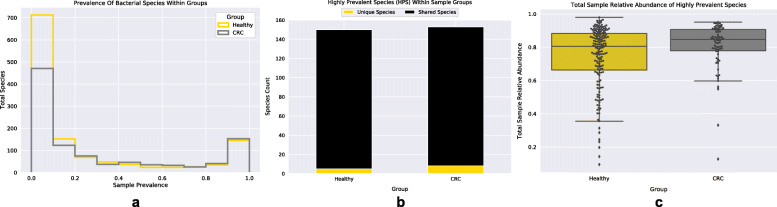
Highly Prevalent and Abundant Species within Groups. **a**. Bi-modal distribution of species prevalence counts across samples within the Healthy and CRC sample groups. **b**. Stacked-bar plot showing the total unique and shared species of the highly prevalent (> 90% prevalence) species within the Healthy and CRC sample groups. **c**. Boxplot of the total sample relative abundance accounted for by the highly prevalent species within groups. Black dots represent individual samples

Species chosen for network construction were selected based on their prevalence, abundance, and ‘importance’. First, the prevalence of each species was calculated across all samples within each group (Fig. 3a). The distributions of bacterial species prevalence counts within groups were found to exhibit a bi-modal distribution with one peak occurring at the 90% prevalence threshold. Going forward we refer to the species found above 90% sample prevalence within groups as the highly prevalent species (HPS). A large majority of species within each group’s HPS were found to be shared (Healthy: 97% and CRC: 95%) (Fig. 3b). The five unique HPS in the Healthy group were: *Hespellia stercorisuis*, *Clostridium saccharolyticum*, *Monoglobus pectinilyticus*, *Streptococcus sp. oral taxon 431*, and *Odoribacter laneus*. The eight HPS unique to the CRC associated group were: *Intestinibacillus massiliensis*, *Prevotella copri*, *Haemophilus parainfluenzae*, *Ruminococcus bicirculans*, *Streptococcus mitis*, *Neglecta timonensis*, *Bifidobacterium catenulatum*, and *Anaerotignum neopropionicum*. Interestingly, *Streptococcus mitis* and *Haemophilus parainfluenzae* are both classified by the eHOMD as oral microbes. The relative abundances of HPS were found to account for the majority (Median = 82%) of a sample’s total relative abundance (Fig. 3c). Moving forward we utilized the union of HPS within groups for network construction. In addition to these highly prevalent and abundant species we wanted to incorporate the species who were both deemed significantly ‘important’ by our RFCs and found in differential abundance. This led to the addition of 8 species (*Parvimonas micra*, *Peptostreptococcus stomatis*, *Gemella morbillorum*, *Fusobacterium nucleatum*, *Streptococcus anginosus*, *Dialister pneumosintes*, *Peptostreptococcus anaerobius*, and *Ruminococcus torques*) to our species group (165 species total) used in network construction. Bacterial association networks were then constructed from the CLR-transformed relative abundance of these selected species (see methods and supplemental information for additional information). Following network construction, we first checked our networks for non-randomness by comparing multiple network properties (average shortest path length, transitivity, and modularity) to those displayed from random networks (see methods). Compared to random networks, the Healthy and CRC networks both exhibited statistically significant (Monte Carlo Simulation; MCS) shorter average shortest path lengths (ASPL) (Healthy and CRC: MCS *p*value< 0.001), higher transitivity (Healthy and CRC: MCS *p*value< 0.001), and higher modularity (Healthy and CRC: MCS *p*value< 0.001) (Table 1). These results indicate that networks constructed displayed properties that were significantly non-random, and that species within networks: are connected to one another through short paths, have positive associations with the neighbors of their neighbors (friends of friends), and form modules (i.e. a group or cluster of species) that are characterized by the majority of associations occurring between species within the same module, and few associations existing with species outside the module.

**Table 1:** Group Network Properties Compared to Random Networks

Network	Nodes	Edges	Density	ASPL	Transitivity	Modularity
CRC	165	324	0.024	*** 1.687	*** 0.379	*** 0.689
Healthy	165	292	0.022	*** 1.554	*** 0.453	*** 0.742

Network properties of Healthy and CRC networks. Both Healthy and CRC networks were found to exhibit significantly shorter Average Shortest Path Lengths (ASPL), higher Transitivity, and higher Modularity then 1000 random networks. Stars (***) denote statistical significance (Monte Carlo simulation pvalue < 0.001)

**Fig. 4:**
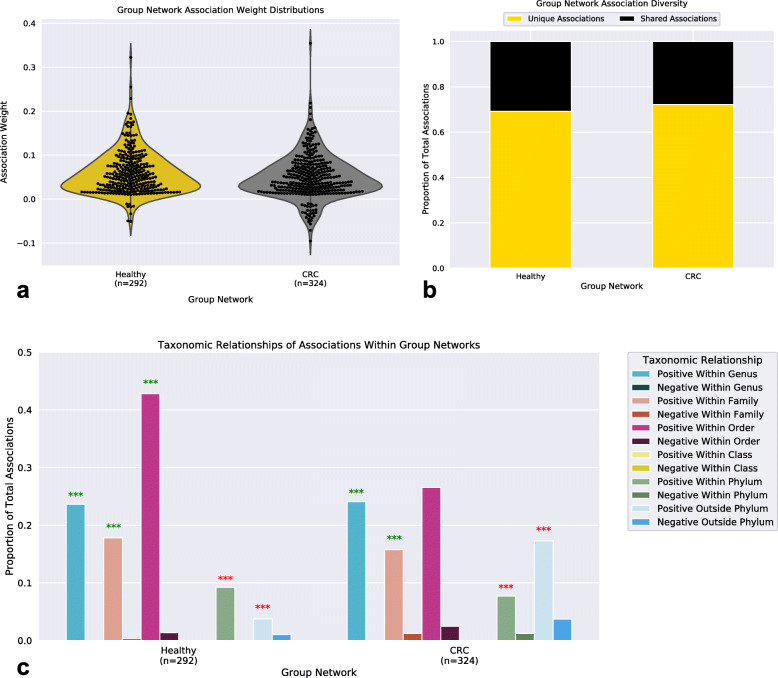
Group Network Associations. **a**. Distribution of bacterial association weights within Healthy and CRC networks. **b**. Stacked-bar plot of the proportion of associations (edges) that are unique and shared between Healthy and CRC networks. **c**. Bar-plots representing the proportion of total associations within the lowest common taxonomic relationship between bacterial species. Stars (***) indicate statistical significance (Monte Carlo simulation pvalue < 0.001), star color (green or red) indicate higher or lower than that found in random networks, respectively

Group networks contained similar distributions of association weights with positive associations being in greater abundance than negative associations (Fig. 4a). Notably, the CRC network contained a greater total of negative associations compared to that found in the Healthy network. Interestingly, 29% of these negative associations involved a species deemed as an oral microbe, whereas within the Healthy network zero negative associations were found to involve oral microbes. Surprisingly, the majority of associations found within networks were unique to that network (Healthy: 69%, CRC: 72%) (Fig. 4b). We hypothesized that this dramatic difference in community structure could reflect changes in the ecosystem and proceeded to analyze the taxonomic relationship between species within networks (see methods) (Fig. 4c). Both networks exhibited significantly (MCS pvalue< 0.05) more positive relationships between species within the same genera (Healthy: MCS pvalue = 0.00099, CRC: MCS pvalue = 0.00099) and family (Healthy: MCS pvalue = 0.00099, CRC: MCS *p*value = 0.00099) compared to those found in a random network (see methods). However, only within the Healthy network did species still have significantly more positive associations with other species from the same order more so than random (Healthy: MCS *p*value = 0.00099, CRC: MCS pvalue = 0.44). The CRC network also exhibited a larger abundance in taxonomically distant (outside phylum) relationships compared to the Healthy network (Healthy: 4%, CRC: 17%), although positive associations between taxonomically distant microbes were still significantly less in Healthy (Within Phylum: MCS pvalue = 0.00099, Outside Phylum: MCS *p*value = 0.00099) and CRC (Within Phylum: MCS pvalue = 0.00099, Outside Phylum: MCS *p*value = 0.00099) than random networks. We next examined the dissimilarity between functional profiles of associating species within the Healthy and CRC networks (Supplemental 6a-c). Interestingly, many of the bacterial associations that are unique to the CRC network were shown to be occurring between species that were functionally dissimilar to one another.

**Fig. 5:**
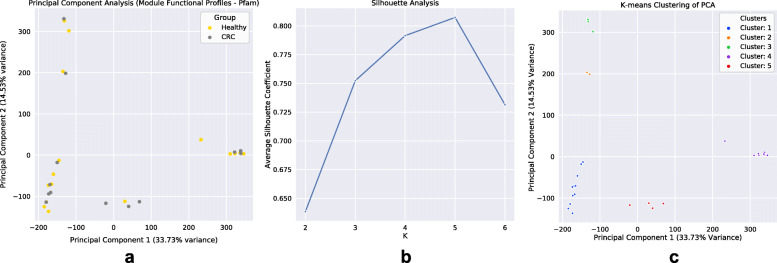
Species Module Functional Clusters Within Networks. **a**. Principal component analysis of module functional profiles. Gold and grey dots represent individual modules from Healthy or CRC networks, respectively. **b.** Silhouette analysis showing K = 5 having the highest average silhouette coefficient. **c**. K-means clustering of the module functional profile PCA using K = 5

**Fig. 6:**
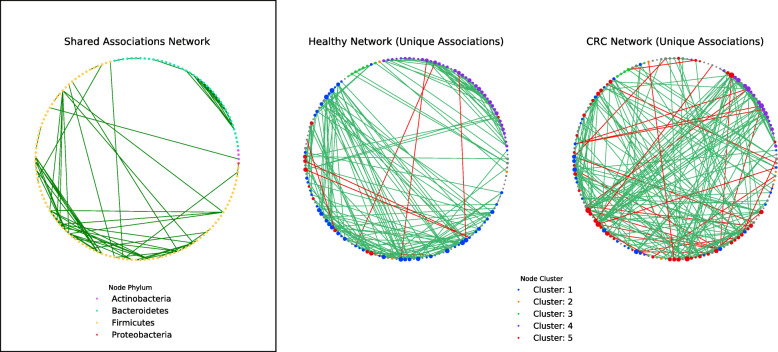
Healthy and CRC Bacterial Association Networks. Bacterial association networks presented in a circular layout. Edge color (green or red) represent positive or negative associations, respectively. Far left network (Shared Associations Network) shows the associations (edges) found in both the Healthy and CRC network. Node color within that network represents the phylum of the species. The two networks on the right are displaying the associations unique only to the Healthy or CRC network. Node color within these networks represent the module cluster this species was found within. Node size is a function of the node’s degree (total associations). For a list of species shown and not shown within networks see supplemental

**Fig. 7:**
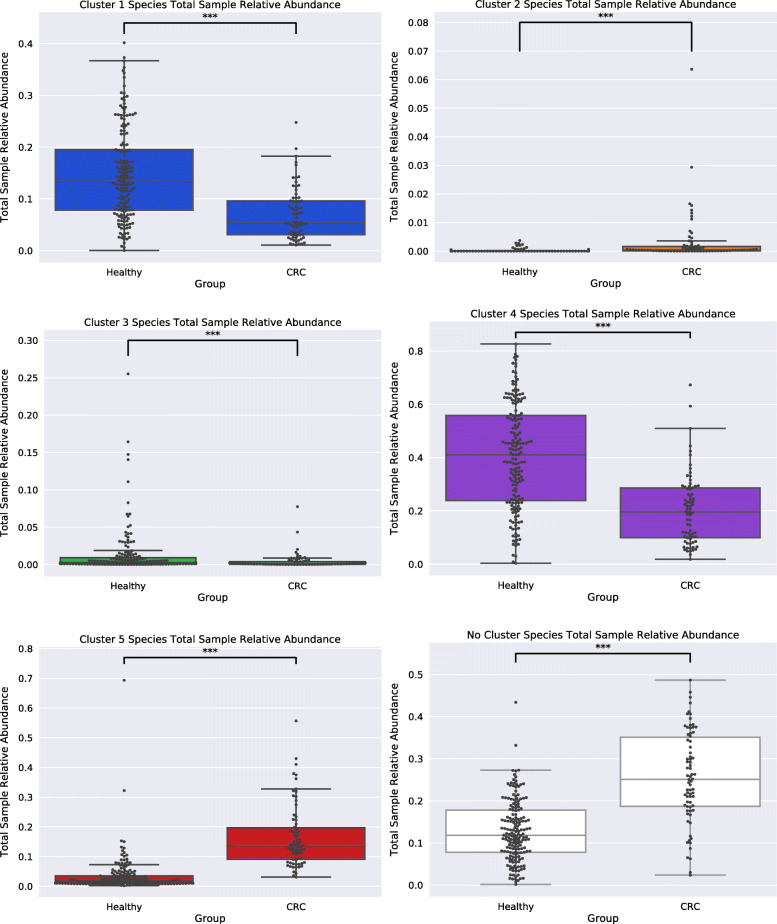
Cluster Species Total Sample Relative Abundance. Boxplots of the total sample relative abundance that all species within each module cluster account for within groups. The species within module clusters 1, 4, and 3 account for a significantly greater total sample relative abundance within the Healthy network compared to the CRC network. The species within module clusters 2 and 5 and no cluster account for a significantly greater total sample relative abundance within the CRC network compared to the Healthy network. Stars (***) indicate statistical significance (MWU pvalue < 0.001)

Considering that our networks exhibited high modularity, and that community functions in microbial environments are driven through polymicrobial synergy [49, 50], we applied a module detection algorithm to our networks, and proceeded to analyze the obtained species modules within our networks (see methods). We first started by comparing the potential functional capabilities of modules by constructing CLR-transformed module functional profiles (see methods). PCA of module functional (protein domain) profiles exhibited large variance (PC1: 33.73%, PC2: 14.53%), and modules appeared to form clusters which contained representation from both groups (Fig. 5a). To define clusters of modules, silhouette analysis was performed which estimated five clusters as the optimal K to use for K-means clustering (Fig. 5b). After module clusters were defined by K-means clustering (Fig. 5c), taxonomic analysis of these clusters was carried out. Across networks, modules that fell within the same cluster were found to be taxonomically similar, excluding cluster 1 and cluster 5 which exhibited a shift in species occupancy where some species found within cluster 1 in the Healthy network were shown to be within cluster 5 in the CRC network, and vice-versa (Supplemental 7a,b,c,g). However, both networks had strong agreement on the species found within clusters 2, 3, and 4. Species within cluster 2 were *only* ‘pathobiont’ (i.e., species that are generally not harmful but contain the capacity to cause disease under particular environmental conditions [51, 52]) oral microbes (Supplemental 7d), whereas cluster 3 was mainly Streptococcus species (Supplemental 7e), and cluster 4 predominantly Bacteroides species (Supplemental 7f). Subsequently, cluster functional analysis was performed to find protein domains, as well as the main roles and sub roles of protein families, which made clusters functionally ‘distinct’ from one another (see methods) (Supplemental 8a-c). Functional capabilities (protein domains and protein family main/sub roles) distinguishing cluster 1 were linked to: cell surface adhesion, counter-conflict strategies, tyrosine recombinases, degradation of polysaccharides, glycosaminoglycan binding, tumor protease inhibition, peroxidase functions, carbohydrate/cellulose binding activities, and amino acid biosynthesis. Cluster 2’s distinguishing functions were linked to: adherence to host cells and extracellular matrix, cellular infection, collagen binding, complement resistance, ornithine/lysine/arginine decarboxylase (tissue putrefaction/polyamine synthesis/acidic environment resistance), metallopeptidases, type V secretion systems, ammonia production, and excretion of poisonous metal ions (copper efflux system), cell envelope, DNA metabolism, fatty acid and phospholipid metabolism, biosynthesis and degradation of surface polysaccharides and lipopolysaccharides. Cluster 3’s distinguishing functions were linked to: mucin binding, zinc scavenging/uptake, cell-surface adhesion, glucose binding/transport, and copper binding, protein and peptide fate/synthesis/secretion, degradation of polysaccharides/carbohydrates, organic alcohols, and acids. Cluster 4’s distinguishing functions were linked to: metal binding, diguanylate cyclase/phosphodiesterase, quorum sensing, carbohydrate-binding, and cysteine/papain proteases, nucleosides and nucleotides, transport and binding proteins, TCA cycle, iron carrying, and the degradation and biosynthesis of surface polysaccharides. Lastly, cluster 5’s distinguishing functions were linked to: aminopeptidases, tripartite tricarboxylate receptors, ethanolamine transportation, starch utilization, and xyloglucan/polysaccharide binding, energy metabolism, amino acids and amines, cation and iron compounds, electron transport, and the biosynthesis and degradation of surface polysaccharides and lipopolysaccharides. The abundance of species utilized for network construction found within each cluster was examined (Healthy: cluster 1 (33%), cluster 2 (2%), cluster 3 (5%), cluster 4 (26%), cluster 5 (7%), no cluster (27%); CRC: cluster 1 (19%), cluster 2 (3%), cluster 3 (3%), cluster 4 (12%), cluster 5 (30%), no cluster (33%)) (Fig. 6). Our findings showed that within the CRC network there was an increase in the total species found within a module of cluster type 2 and 5 and a reduction of species in cluster type 1, 3, and 4 compared to the Healthy. These results are also reflected in our findings of a statistically significant change in the total sample relative abundance that species within clusters accounted for between groups (Cluster 1: MWU pvalue = 4.29e-12; Cluster 2: MWU pvalue = 3.16e-16; Cluster 3: MWU pvalue = 0.0002; Cluster 4: MWU pvalue = 2.62e-13; Cluster 5: MWU *p*value = 2.81e-29; No Cluster Species: MWU pvalue = 4.40e-17) (Fig. 7). Moreover, the majority of negative associations within networks (Healthy: 100%, CRC: 96%) were found to occur between species that occupy modules within different cluster types (Supplemental 9). Interestingly, only within the CRC network did an intra-cluster negative association arise between species of cluster 1 where a reduction in species membership and abundance was also exhibited.

### Influential bacterial species within networks

Finally, we examined which species potentially have the greatest influence on the structure of our networks, and therefore possibly within the ecosystem as well, by identifying ‘Hub’ nodes. ‘Hub’ nodes are species with many associations that serve as a central point of connection between many other species [53, 54]. Most modules within networks (Healthy: 84.6%; CRC: 87.5%) were found to be disassortative with respect to node degree (Supplemental 10) suggesting that ‘Hub’ species existed within these modules [53]. We proceeded to identify ‘Hub’ species by selecting the species with the largest degree centrality within all modules exhibiting a degree assortativity below zero (see methods). In total, 22 unique ‘Hub’ species were identified, and of these ‘Hubs’ only two, *Bacteroides fluxus and Bacteroides pectinophilus,* were shared between Healthy and CRC networks. We noted that *Bacteroides fluxus* and *Bacteroides pectinophilus* also maintained their position as ‘Hubs’ within the same module cluster type (Cluster 4 and Cluster 1, respectively) across networks (Supplementary 11a,b). Interestingly, only within the CRC network were oral microbes, *Peptostreptococcus stomatis* and *Streptococcus parasanguinis*, designated as ‘Hub’ nodes. The module *Peptostreptococcus stomatis* is a ‘Hub’ within is particularly fascinating as it is the only CRC cluster 2, ‘pathobiont’ cluster, module where all species are both oral microbes (*Gemella morbillorum*, *Parvimonas micra*, and *Dialister pneumosintes*) and found to be significantly elevated in relative abundance. Moreover, *Anaerotruncus colihominis*, a ‘Hub’ species only within the Healthy network, was found to be negatively associated with *Gemella morbillorum* within this module in the CRC network (Supplementary 12).

## Discussion

In this study, WGS data available from healthy and late-stage colorectal cancer subjects were utilized in conjunction with community profiling and network inference techniques to better understand the alterations in bacterial community ecology that have occurred within the late-stage cancer-associated human gut microbiome. Our study uncovered key distinctions in both the bacterial species and genomic functional capabilities that were different between the two communities, suggesting an overgrowth of potentially pathogenic species classified as oral microbes. We also observed a dramatic difference in bacterial community structure which we believe to be due to an alteration in bacteria partner selection in response to probable ecosystem changes occurring within the CRC-associated gut microbiome.

Our study showed that the CRC gut microbiome contained a significantly higher bacterial diversity. This higher diversity was *somewhat* unexpected since a high bacterial diversity is regularly associated with the healthy gut microbiome [55], and previous studies have described a lower diversity within the CRC gut microbiome [4, 26], although, these findings are still in contention as other studies have also found a higher bacterial richness [56]. In either case, this discrepancy in species diversity estimations between studies could be due to differences in the sequence data type (amplicon vs shotgun) used as 16S rRNA data is known to highly skew estimates of bacterial diversity [57]. We hypothesized that this higher species diversity was due to the formation (or expansion) of a bacterial niche in the cancer-associated ecosystem, most likely caused by the presence of the tumor mass. Any bacterial species existing closely to, or within, the tumor microenvironment (TME) niche would be exposed to a hostile environment characterized by low oxygen, high acidity, and an abundance of oxidative stressors [58, 59]. These environmental conditions are in part created by the altered metabolism of tumor cells which would lead to the reduction in the typical proteins, carbohydrates, and lipids available (nutrient scarcity) in the surrounding microenvironment [60–63]. Tumor cells will also scavenge for any additionally needed resources by degrading the extracellular matrix (ECM), and cannibalizing the surrounding necrotic intestinal tissue to fuel their metabolism [64]. These degradation products could provide certain microbiota capable of utilizing them a rich assortment of free resources including amino acids, membrane proteins, phospholipids, and some sugars. As our CRC samples were obtained from late-stage cancer subjects, this TME niche could be widespread across the colon having repercussions for even microbes not involved in the colonization of this niche. Our findings from using machine learning, differential abundance testing, and network inference point towards species capable of filling this niche, functions likely to promote its formation, and the potential impact that the creation of this niche has on the gut microbiota.

Species differential abundance testing between groups found 174 species significantly reduced and 10 species significantly elevated in relative abundance within the CRC-associated gut microbiome compared to the Healthy gut microbiome. Of the 10 species, six (*Parvimonas micra*, *Peptostreptococcus stomatis*, *Gemella morbillorum*, *Fusobacterium nucleatum*, *Streptococcus anginosus*, and *Peptostreptococcus anaerobius*) were previously found elevated in relative abundance by the research study that generated the data analyzed here [31]. However, we additionally found *Dialister pneumosintes*, *Streptococcus sp. KCOM 2412* (*Streptococcus periodonticum*), *Ruminococcus torques*, and *Filifactor alocis* as being significantly elevated in relative abundance within the CRC sample group. This discrepancy in findings is most likely due to differences in both read mapping and species relative abundance calculations. That study mapped reads to the All-Species Living Tree Project (LTP) of the SILVA database [65] assigning taxonomy to the species which provided the lowest E-value, and calculated species relative abundances as the number of reads assigned to the species divided by the total number of aligned reads within the sample. In contrast, we mapped reads to a comprehensive collection of bacterial reference strain genomes downloaded from RefSeq [66], and calculated species relative abundances utilizing an accurate probabilistic framework [67]. To our knowledge, this is the first time *Filifactor alocis* has been shown to have elevated relative abundance within CRC. *Filifactor alocis*, previously known as *Fusobacterium alocis*, is a gram-positive obligate anaerobe that has routinely been discovered in periodontitis and endodontic infections and is described as an excellent marker organism for periodontal disease [40, 68, 69]. Interestingly, all 10 of the species found significantly elevated in relative abundance within CRC were classified as oral microbes, and despite normally existing within the Healthy gut microbiome these species are considered ‘pathobionts’ as they have numerous associations with infections [37, 70] and even CRC [56, 71–75]. Many of these species also have been previously shown to exist in close association with colonic tumor tissues [72] and possess the capability to colonize the TME niche as they are: anaerobic [76], regularly form biofilms together [39, 77], and exhibit asaccharolytic metabolism [76]. Since oral microbes exhibit an asaccharolytic metabolism they target peptides and amino acids for their digestion [76] and in doing so produce ammonia which would raise the local pH helping their colonization within the acidic TME. In this way, these species would be optimized for growth in the hostile TME niche. Outside of just these 10 oral species, we also uncovered a significantly higher richness of bacteria classified as oral microbes within the CRC gut microbiome. This finding suggests that oral microbes have become increasingly more capable of colonizing the gut within the CRC-associated ecosystem.

Interestingly, of the few bacterial community functions (Pfams and TIGRFAMS) found in differential abundance between the CRC and Healthy gut microbiomes, many could precipitate cancer progression, or aid in the colonization of the TME niche. Multiple protein functions found to be significantly reduced within the CRC gut microbiome were tied to threonine biosynthesis. Threonine is an essential amino acid; therefore, it must be provided exogenously from the gut microbiota’s metabolism [78]. It is also an important amino acid in the production of short chain fatty acids (SCFAs) since it can be utilized for the formation of acetate, butyrate, or propionate [79]. Interestingly, of the 174 species found significantly reduced in CRC many are from genera (*Lactobacillus*, *Bacteroides*, *Bifidobacteria*, *Clostridium*, *Eubacterium*, etc.) shown to be linked to the production of SCFAs [80–82]. The reduction in the enzymatic capability to synthesize threonine could drive tumor progression as SCFAs (e.g., butyrate) have been shown to have anti-oncogenic and anti-inflammatory properties [83]. Of the functions found significantly elevated in relative abundance in the CRC gut microbiome many were tied to adhesins and invasins. These protein functions would allow bacteria to adhere to epithelial cells, especially those that are being sloughed off the intestinal wall, to gather nutrients. They would also assist in the invasion of the intestinal barrier which would drive inflammation and could cause DNA damage thereby inducing unwanted cellular mutation. For example, FadA, an adhesin found significantly elevated in relative abundance, is unique to the oral lineage of *Fusobacterium nucleatum’s phylum (Fusobacteria)* and has previously been shown to promote binding and invasion into host epithelial cells [84], as well as driving cancer initiation [85, 86]. Additionally, we found a significantly elevated relative abundance of a protein function linked to proline iminopeptidase (PIP), an enzyme that catalyzes the release of proline residues from peptides. Proline is an important stress substrate in cancer metabolism as it is utilized in many critical functions related to apoptosis, autophagy, and nutrient/oxygen deprivation [87]. Tumor cells can harvest the proline they require by metabolizing collagen contained within the extracellular matrix (ECM), as nearly 25% of the collagen is proline [88]. Interestingly, in our study a few of the oral species found significantly elevated in relative abundance (*Peptostreptococcus stomatis*, *Gemella morbillorum*, *Parvimonas micra*, and *Dialister pneumosintes*) were shown to form a network module with the functionally distinct capability to bind and degrade collagen.

Only a few associations were shared between the bacterial association networks for the two sample groups which suggested there was a large difference in the bacterial community structure within Healthy and CRC-associated gut microbiomes. Part of the difference in community structure occurring within the CRC-associated gut microbiome is due to positive associations forming less between species that were taxonomically similar, and more between functionally dissimilar species compared to those found in the Healthy gut microbiome. Moreover, we found a greater number of negative associations within the CRC network, and in many of these negative associations an oral microbe was found to be involved, whereas, in the healthy network no such negative associations with oral microbes were occurring. This suggests that competitive exclusion between taxonomically and functionally similar species within the CRC-associated gut microbiome has increased, and oral microbes have become more competitive within this ecosystem. Additionally, as oral microbes are also found to be present within the Healthy gut microbiome, but negative associations against oral microbes were not, we hypothesized that the native microbiota has shifted towards utilizing similar resources to those targeted by oral microbes within the CRC gut microbiome. Our analysis of species modules within networks reflects this notion. Using PCA and K-means clustering, species modules within networks were found to fall into one of five distinct clusters depending on their functional capabilities. However, both Healthy and CRC networks contained representation (at least one module) within all clusters suggesting the niches that these clusters target are maintained across Healthy and CRC-associated gut microbiomes in some capacity. Yet, despite cluster retention, there was a dramatic shift in both the proportion of total species and the total sample relative abundance certain clusters accounted for within networks. For example, within the Healthy network we found clusters functionally geared towards amino acid biosynthesis, carbohydrate degradation, protein binding/uptake, and tumor inhibition contained a greater number of species and represented a larger total sample relative abundance. Whereas, in the CRC network we observed a species shift towards forming modules functionally equipped for protein degradation, amino acid uptake, biosynthesis and degradation of surface polysaccharides and lipopolysaccharides, and ethanolamine utilization. Interestingly, *Klebsiella* species have been tied to ethanolamine usage in the healthy gut [89, 90] and were found in reduced relative abundance in the CRC gut microbiome, suggesting that these species were potentially outcompeted. In any case, this shift in species cluster membership and cluster total sample relative abundance suggests that the bacterial community structure has been reorganized to aid in the formation of modules of specific cluster types that contain functional capabilities better suited for life in the CRC-associated gut environment.

As mentioned previously, one module cluster (cluster 2) drew our attention as it was comprised solely of ‘pathobiont’ oral species and contained distinct functions which would allow these species to not only flourish within the TME niche but aid in cancer progression. These functions included: adherence to host cells and extracellular matrix, collagen-binding, complement resistance, ornithine/lysine/arginine decarboxylase (tissue putrefaction/ polyamine synthesis/acidic environment resistance), metallopeptidases, type V secretion systems, ammonia production, excretion of poisonous metal ions (copper efflux system), DNA metabolism, fatty acid and phospholipid metabolism, and biosynthesis and degradation of surface polysaccharides and lipopolysaccharides. Despite a module of this cluster type existing within the Healthy network, all species existing within the CRC module (*Peptostreptococcus stomatis*, *Gemella morbillorum*, *Parvimonas micra*, and *Dialister pneumosintes*) were found to be significantly elevated in relative abundance. It is also important to note that this module in the CRC network grew with the addition of another oral species, *Dialister pneumosintes*. Which suggests these oral species are indeed thriving in the CRC-associated gut microbiome and through their metabolic actions potentially driving tumor progression. It could be prudent to preemptively target *Peptostreptococcus stomatis* for elimination from the gut microbiome as it was the ‘hub’ species within the module. By doing so this could lead to the dissipation of the associations between these species and potentially dampen tumor progression. In any case, future in vivo studies should be performed to elucidate the extent that polymicrobial synergy between these species contributes to tumorigenesis.

## Conclusion

In summary, our analysis of whole-genome shotgun sequenced fecal samples provided from a large cohort of late-stage colorectal cancer and healthy subjects revealed key differences in the bacterial community within Healthy and CRC-associated gut microbiomes. We showed a higher species diversity exists within the CRC-associated gut microbiome that is potentially due to the formation of a tumor-associated niche, and this niche is most likely occupied by species originating from the oral cavity. Moreover, we highlighted *Peptostreptococcus stomatis* as an influential ‘hub’ node within a ‘pathobiont’ oral species module where every species within the module were found in elevated relative abundance in CRC. Our results also indicated that tumor presence influences the reorganization of the native bacterial community structure to aid in the formation of modules that contain functional capabilities better suited for life in the CRC-associated gut environment.

## Methods

### Data acquisition and cohort description

For this study, 252 whole-genome shotgun sequenced fecal samples were retrieved from DDBJ Sequence Read Archive (DRA) under the bioproject ID PRJDB4176 [31]. The original study population of this cohort consisted of healthy and early/advanced colorectal cancer stage patients who were undergoing total colonoscopy at the National Cancer Center Hospital, Tokyo, Japan. Fecal samples were collected immediately following the first defecation after a bowel-cleansing agent was administered orally. Cancer patients who had or were thought to have hereditary disease, an inflammatory bowel disease, an abdominal surgery history, or whose stool samples were insufficient for data collection were excluded from the original study. Samples chosen to be utilized within this study came from 178 healthy and 74 late-stage (52 stage III / 22 stage IV) colorectal cancer (CRC) subjects. Sample groups had comparable male to female frequencies (Healthy: 56.18/43.82; CRC: 58.11/41.89) (Supplemental 13a) and subject ages (Healthy median age: 62; CRC median age: 61) (Supplemental 13b). For additional information on all samples used in this study see supplemental file.

### Data pre-processing

Reads were trimmed with Trimmomatic [91] (version 0.36) utilizing a 4:15 sliding window approach where a read is clipped once the average quality score within a sliding window of 4 base pairs drops below a quality score of 15. Afterwards, reads from human origin were filtered by utilizing Bowtie2 [92] (version 5.4.0, −-very-sensitive setting) and the GRCh38.p12 human genome [93].

### Species level community taxonomic profiling

For bacterial community taxonomic profiling of WGS reads we elected to utilize a reference-based mapping approach. Sample reads were mapped to a reference database of 10,839 bacterial reference strain genomes downloaded from RefSeq [66] utilizing Bowtie2 (version 5.4.0, settings: --very-sensitive --reorder --mp 1,1 --rfg 1,1 -k 1000 –score-min L,0,-0.1). In total over 3.5 billion (3,515,063,526) reads were mapped. Next, a probabilistic framework based on a mixture model [67, 94] was used to analyze the read mapping information to estimate the relative copy number of each reference genome in a sample. This framework used an Expectation-Maximization (EM) algorithm to optimize the log-likelihood function associated with the model. We have previously shown our EM algorithm to be highly accurate in its species relative abundance estimation capabilities [95]. Any bacterial strain found within a sample in less than 1e-5 relative abundance was considered to be noise and their abundance was dropped to 0. Bacterial strain-level assignments were rolled back to species-level assignments (by using accession and tax ids with NCBIs taxonomic assignments), and relative abundances were summed to produce bacterial species genome relative abundances. Principal components analysis was performed using Scikit-learn (version 0.23.2). Before PCA, species relative abundances within the sample-taxa matrix were first Centered Log-Ratio (CLR) transformed (all zero values were replaced with 1e-10 before transformation). CLR-transformation [24] is defined as:

$$ \mathrm{clr}\left(\mathrm{x}\right)=\left[\ln \frac{{\mathrm{x}}_1}{\mathrm{g}\left(\mathrm{x}\right)},\ln \frac{{\mathrm{x}}_2}{\mathrm{g}\left(\mathrm{x}\right)}\dots, \ln \frac{{\mathrm{x}}_{\mathrm{D}}}{\mathrm{g}\left(\mathrm{x}\right)}\right] $$

where (x) is the vector of species abundances within each sample and (D) is the total number of species. The geometric mean of vector (x) is defined as:

$$ g(x)=\sqrt[D]{x_1\times {x}_2\times \dots {x}_D} $$

### Random Forest analysis

CLR-transformed species relative abundances were analyzed using the Random Forest Classifier (RFC) package from Scikit-learn [96]. Random forests were trained and tested with a 70% training and 30% testing sample split and 100 trees per forest. One-hundred RFCs were constructed in order to deem a species as significantly ‘important’. First, a ‘random’ feature was created from randomly selected CLR-transformed species sample relative abundances to assist in the selection of significantly ‘important’ species, as default importance measurements from random forest classifiers are known to be biased [35]. Next, the importances (Gini importance) for each species provided from all 100 RFCs was compared to those of the 100 ‘random’ feature importances. Only species with statistically significant higher ‘importance’ according to a Mann-Whitney U test and Benjamini-Hochberg (FDR) multiple testing correction (MWU-FDR: qvalue< 0.05) were deemed significantly ‘important’. The AUC (Area Under the Receiver-Operator Curve) and Classification Accuracy (Jaccard index) were both utilized to measure the accuracy of trained forests. The AUC is an estimator of true and false positive prediction rates of our RFC, whereas the Classification Accuracy computes subset accuracy (where a prediction for a set of labels must *exactly* match those from the known true corresponding label set).

### Bacterial species diversity analysis

To measure the diversity of species found within each sample, total bacterial richness (total species found in a sample) and the Shannon index [97] were calculated. The Shannon index is calculated as:

Shannon Index (H) = $$ -{\sum}_{i=1}^D{P}_i\cdotp \mathit{\ln}{P}_i $$

where (D) is the total number of species, and (P_i_) is the proportion of that species within the sample.

### Differential relative abundance of species

Species relative abundances within the sample-taxa matrix were first CLR-transformed (all zero values were replaced with 1e-10 before transformation). Mann-Whitney U test and FDR correction were utilized to test for significant species relative abundance differences between groups. Only species with a qvalue < 0.05 and a sample prevalence greater than 10% within at least one group were deemed truly differentially abundant.

### Bacterial species functional profiles

Gene prediction was performed on all bacterial reference strain genomes utilizing Prodigal [98] (version 2.6.3). All protein sequence translations for genes output by prodigal were provided to InterProScan [99] (version 5.39–77.0) to find matches for protein domains and protein families against the Pfam [48] (version 32.0) and TIGRFAM [47] (version 15.0) databases, respectively. All Pfams and TIGRFAMS found within genomes were counted and then counts were normalized (by total) producing relative abundances. Species functional profiles were created separately for Healthy and CRC groups. This was performed by weighing strain functional profiles by strain average abundance within a group and then summing the strain functional profiles together, followed by re-normalization (by total).

### Sample functional profiling

To explore the bacterial community functional capabilities contained within each sample, a simplified annotation format file (SAF) containing the bacterial chromosomal coordinates of features (either Pfams or TIGRFAMs) for all strains was created. Next, the SAF was provided to FeatureCounts [100] (Subread package 2.0.0) to find all features contained within the sample reads. Lastly, the counts of features were subsequently length normalized, summed, then re-normalized (by total) to create a sample functional profile. Function (Pfams or TIGRFAMS) relative abundances within the sample-function matrix were first CLR-transformed (all zero values were replaced with 1e-10 before transformation). Mann-Whitney U test and FDR correction were utilized to test for significant function relative abundance differences between groups. Only functions with a qvalue < 0.05 were deemed significantly different.

### Species selection for association network construction

Species selected for network inference were either highly prevalent/abundant species (the union of species exhibiting > 90% sample prevalence within both groups), or species that were deemed as both significantly ‘important’ by random forests and differentially abundant. In total there were 165 species selected for network construction.

### Bacterial association network inference

For each sample group, a bacterial association network was constructed from CLR-transformed sample-taxa matrix of that group using a Gaussian Graphical Model (GGM) framework. For each group, a sparse precision matrix (Ω) was computed using the huge [101] package in R, and this matrix formed the adjacency matrix of the association network. The stability approach to regularization (StARS) method [102] was utilized to choose the tuning parameter (ρ) in the l1-penalty model for sparse precision matrix estimation. The partial correlation matrix, *P,* was calculated as:

$$ {P}_{\left[i,j\right]}=\frac{-{\varOmega}_{\left[i,j\right]}}{\sqrt{\varOmega_{\left[i,i\right]}\times {\varOmega}_{\left[j,j\right]}}} $$

Finally, any associations below a magnitude of 0.01 within the partial correlation matrix was treated as statistical noise and removed.

### Network topology comparison

For each network, the following properties were computed using NetworkX [103] (version 2.4): average shortest path length (ASPL), transitivity, and modularity. The ASPL (α) was calculated as:

$$ \alpha ={\varSigma}_{s,t\in L}\frac{D\left[s,t\right]}{n\left(n-1\right)} $$

where (L) is the set of nodes in the graph (G), the shortest path between the nodes (s) and (t) is D [s,t], and (n) is the total number of nodes in (G). The transitivity (T) of a network was calculated as:

$$ T=3\frac{Total\ triangles}{Total\ triads} $$

in which triangles are considered a subset of three nodes within a network where each node is adjacent to all other nodes within the subset, and triads are connected triples (i.e. three nodes abc where edges (a,b) and (b,c) exist and the edge (a,c) can be present or absent). Transitivity is the fraction of all possible triangles present in the graph and is a measurement of node clustering. Finally, the modularity (Q) [104] of a network was calculated as:

$$ Q=\sum \limits_{c=1}^n\left[\frac{L_c}{m}-{\left(\frac{k_c}{2m}\right)}^2\right] $$

where (n) is all modules of a graph partition, (c) is an individual module from the partition, (m) is the total number of edges of the graph (G), (L_c_) is the total intra-module edges, and (k_c_) is the sum of edges of all nodes within module (c). Networks were first partitioned into modules before modularity could be calculated (for module detection see Module Functional Profiles below). Monte Carlo simulations were utilized to test networks for non-randomness where 1000 random (G_n,p_) networks were created, using NetworkX, and network properties (ASPL, transitivity, and modularity) were measured and used to produce pvalues for group network properties. For the creation of random networks (n) was equal to the group network of interest’s node total and (p) the network density.

### Taxonomic relationship analysis of species associations

For each association, the lowest common taxonomic relationship between species was characterized by using the NCBI taxonomic assignments. Monte Carlo simulations were utilized to test for significance and produce pvalues. First, 1000 random (G_n,m_) networks were produced, using NetworkX, for comparison to each group network. Within these networks (n) was equal to the group network node total, and (m) the total edges (associations) within group networks. Next, species names and association weights from group networks were randomly assigned to nodes and edges within each random network. Lastly, the total of each lowest common taxonomic relationship between nodes in each random network were computed and compared to those found within group networks.

### Module functional profiles

Species modules were first detected within networks utilizing an asynchronous label propagation algorithm [105] for module detection. The module detection algorithm was allowed to partition the graph into modules 100 times. The modules produced from the partition resulting in the highest ‘performance’ were kept for subsequent analyses. Performance (p) is calculated as:

$$ p=\frac{a+b}{t} $$

where (a) is the total intra-module edges, (b) the total inter-module non-edges, and (t) is the total potential edges. Following module detection, module functional profiles were created by weighing the species functional profile (Pfam or TIGRFAM) of each species within a module by that species mean relative abundance within a group (Healthy or CRC), and then re-normalizing by total.

### Module cluster functional analysis

Module functional profiles were CLR-transformed before PCA. To find clusters, modules were partitioned by performing K-means clustering, from Scikit-learn, on the PCA. Silhouette analysis, from Scikit-learn, was used to find the optimal K for K-means clustering. Silhouette coefficients (SC) range from [− 1,1] where a positive SC near 1 indicates that a module within our PCA is far away from neighboring clusters and a high average silhouette score is indicative of well-defined clusters. After clusters were defined, the distinct functionality of clusters was examined. First, PCA was run in a pairwise fashion on the modules from each cluster to find the most important functional features (Pfams or TIGRFAMS), which made a cluster distinct from every other cluster. Across all PCAs, the features which separated each cluster along the first principal component exhibiting an importance above a magnitude of 0.01 were noted and summed. Afterwards the top 100 TIGRFAMS with the highest importance within each cluster were selected, and the main and sub roles of each TIGRFAM elucidated. TIGRFAM main and sub role abundance importances were created by summing the importances of all TIGRFAMS that were assigned to that main and sub role then normalizing by total. Lastly, the top 10 Pfams with the highest total importance were utilized for a more in-depth inspection into a cluster’s distinct functionality.

### Node centrality ‘hub’ analysis

Degree centrality was used to find bacterial ‘Hubs’ within modules by choosing the species with the most associations (edges) within a module. Only ‘Hubs’ from module sub graphs that exhibited disassortative mixing in respect to degree (degree assortativity < 0), as measured by NetworkX, were selected for examination.

### Statistical significance testing

A two-tailed nonparametric t-test (Mann-Whitney U test) [106] was used to compare groups for statistical significance. Benjamini-Hochberg (False discovery rate; FDR) [107] was used for multiple testing correction.

## Supplementary Information

**Additional file 1.**

## Data Availability

All data and scripts used in this study can be found at https://github.com/Markloftus/CancerMicrobiome.

## Abbreviations

CRC: Colorectal Cancer
TME: Tumor Microenvironment
RFC: Random Forest Classifier
eHOMD: expanded Human Oral Microbe Database
CLR: Centered Log-Ratio
EM: Expectation-Maximization.
GGM: Gaussian Graphical Model
MWU: Mann-Whitney U Test
FDR: False Discovery Rate
