# Supplementary material for: Bacterial community structure alterations within the colorectal cancer gut microbiome
Source: BMC Microbiol. 2021 Mar 31;21:98. doi: 10.1186/s12866-021-02153-x (PMC8011136; doi:10.1186/s12866-021-02153-x)
Supplement: Supplementary file 1 — Additional file 1. [file 12866_2021_2153_MOESM1_ESM.docx]

**Supplemental Information**

**Simpson index of Diversity:**

The Simpson index of species diversity within samples was calculated as:

**Simpson index of Diversity** = (1 – Dominance)

**Dominance** = $\frac{\sum n(n-1)}{N(N-1)}$

Where (n) is the count of each species in a sample and (N) is the total sum of species counts within a sample. To convert species relative abundances to counts samples relative abundance profiles were multiplied by one million.

**Bacterial Association Networks:**

List of bacteria not shown in network illustrations due to them having a degree of zero (0) in both Healthy and CRC networks:

- *Prevotella stercorea, Holdemanella biformis, Clostridium saccharolyticum,*

*Intestinibacter bartlettii, Hespellia stercorisuis, Intestinibacillus massiliensis, Bacteroides zoogleoformans, Bifidobacterium catenulatum, Prevotella copri, Fusobacterium nucleatum, Peptostreptococcus anaerobius, Ruminococcus torques, Lachnoanaerobaculum saburreum, Streptococcus anginosus, Clostridium_sp_SY8519, Ruminococcus lactaris, Odoribacter laneus,*

*Parabacteroides johnsonii*

*
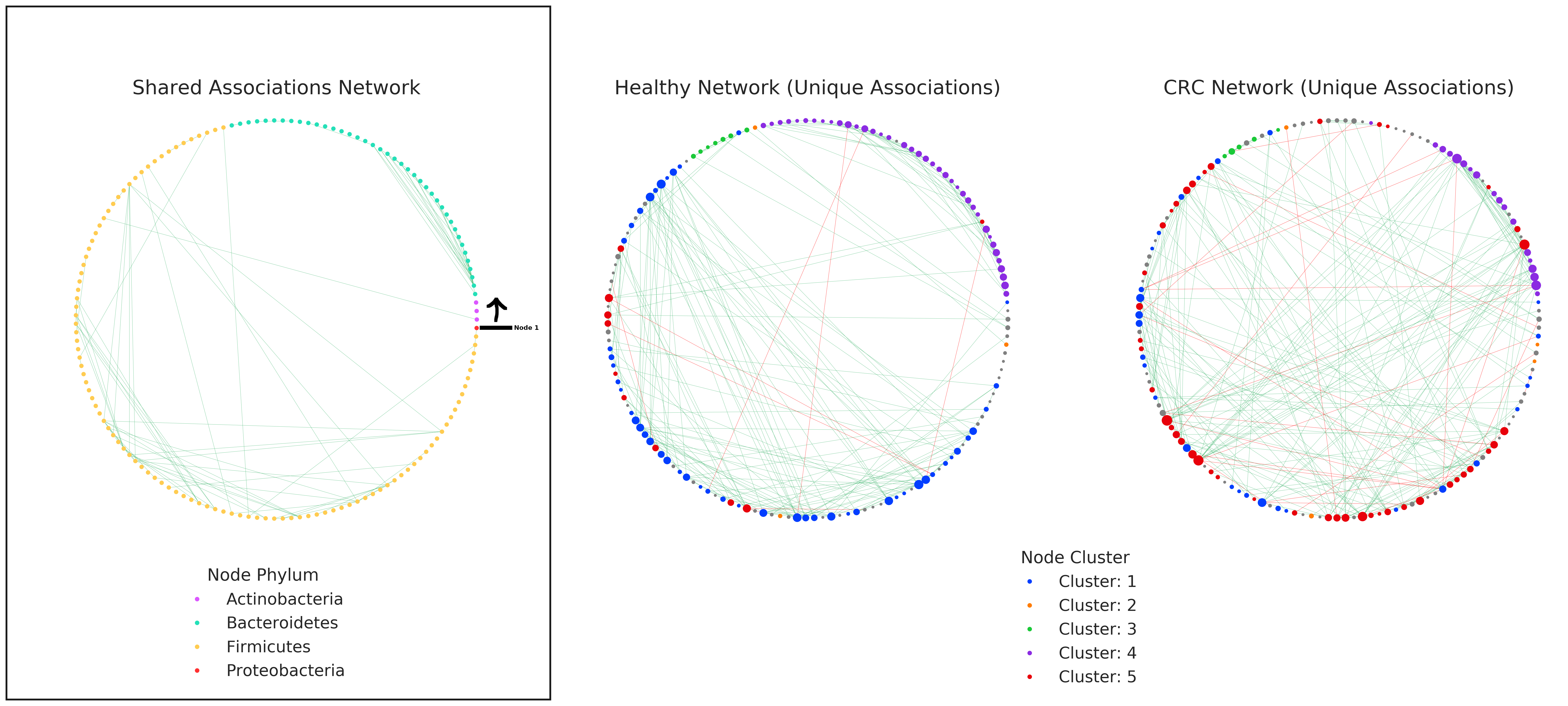
*

*Bacteria shown starting at node 1 and going counterclockwise:*

- *Schaalia_odontolytica, Collinsella_aerofaciens, Eggerthella_lenta, Bacteroides_faecichinchillae, Bacteroides_coprophilus, Bacteroides_caecimuris, Bacteroides_fluxus, Bacteroides_vulgatus, Bacteroides_ovatus, Bacteroides_thetaiotaomicron, Bacteroides_fragilis, Bacteroides_helcogenes, Bacteroides_cellulosilyticus, Bacteroides_plebeius, Bacteroides_nordii, Bacteroides_stercoris, Bacteroides_barnesiae, Bacteroides_coprocola, Bacteroides_salanitronis, Bacteroides_pyogenes, Bacteroides_caccae, Bacteroides_salyersiae, Bacteroides_uniformis, Bacteroides_dorei, Bacteroides_heparinolyticus, Mediterranea_massiliensis, Barnesiella_intestinihominis, Barnesiella_viscericola, Coprobacter_fastidiosus, Coprobacter_secundus, Butyricimonas_faecalis, Culturomica_massiliensis, Odoribacter_splanchnicus, Paraprevotella_xylaniphila, Prevotellamassilia_timonensis, Alistipes_putredinis, Alistipes_shahii, Alistipes_senegalensis, Alistipes_obesi, Alistipes_finegoldii, Parabacteroides_distasonis, Parabacteroides_sp_CT06, Parabacteroides_goldsteinii, Gemella_morbillorum, Granulicatella_adiacens, Lactobacillus_rogosae, Streptococcus_sp_FDAARGOS_192, Streptococcus_mitis, Streptococcus_sp_oral_taxon_431, Streptococcus_sp_A12, Streptococcus_salivarius, Streptococcus_parasanguinis, Christensenella_minuta, Butyricicoccus_pullicaecorum, Clostridium_phoceensis, Clostridium_sporogenes, Hungatella_hathewayi, Lactonifactor_longoviformis, Massilioclostridium_coli, Mordavella_sp_Marseille-P3756, Emergencia_timonensis, Mogibacterium_diversum, Eubacterium_ramulus, Eubacterium_ventriosum, Eubacterium_plexicaudatum, Eubacterium_eligens, Anaerobutyricum_hallii, Anaerostipes_hadrus, Anaerotignum_lactatifermentans, Anaerotignum_neopropionicum, Bariatricus_massiliensis, Blautia_sp_N6H1-15, Blautia_schinkii, Blautia_producta, Blautia_hansenii, Ruminococcus_gnavus, Blautia_obeum, Blautia_hydrogenotrophica, Butyrivibrio_crossotus, Coprococcus_comes, Coprococcus_eutactus, Dorea_formicigenerans, Dorea_longicatena, Faecalicatena_contorta, Fusicatenibacter_saccharivorans, Johnsonella_ignava, Clostridium_citroniae, Clostridium_asparagiforme, Clostridium_glycyrrhizinilyticum, Clostridium_bolteae, Clostridium_symbiosum, Clostridium_scindens, Lachnoclostridium_phocaeense, Lachnoclostridium_sp_YL32, Marvinbryantia_formatexigens, Merdimonas_faecis, Roseburia_hominis, Roseburia_intestinalis, Roseburia_inulinivorans, Roseburia_faecis, Sellimonas_intestinalis, Tyzzerella_nexilis, Lachnospiraceae_bacterium_GAM79, Eubacterium_rectale, Lachnospiraceae_bacterium_Choco86, Oscillibacter_sp_PEA192, Clostridioides_difficile, Peptostreptococcus_stomatis, Acetivibrio_ethanolgignens, Agathobaculum_desmolans, Anaerotruncus_colihominis, Angelakisella_massiliensis, Faecalibacterium_prausnitzii, Flavonifractor_plautii, Fournierella_massiliensis, Gemmiger_formicilis, Negativibacillus_massiliensis, Neglecta_timonensis, Phocea_massiliensis, Provencibacterium_massiliense, Pseudoflavonifractor_capillosus, Ruminococcus_bicirculans, Ruminococcus_faecis, Ruminococcus_callidus, Ruthenibacterium_lactatiformans, Subdoligranulum_variabile, Clostridium_leptum, Clostridium_methylpentosum, Intestinimonas_butyriciproducens, Monoglobus_pectinilyticus, Bacteroides_pectinophilus, Clostridiales_bacterium_CCNA10, Absiella_dolichum, Clostridium_innocuum, Clostridium_saccharogumia, Faecalitalea_cylindroides, Holdemania_massiliensis, Merdibacter_massiliensis, Solobacterium_moorei, Traorella_massiliensis, Erysipelotrichaceae_bacterium_GAM147, Phascolarctobacterium_succinatutens, Dialister_pneumosintes, Veillonella_dispar, Parvimonas_micra, bacterium_LF-3, Haemophilus_parainfluenzae*

**Study Samples:**

Below are the 252 Healthy/CRC whole-genome shotgun sequenced fecal samples which were retrieved from DDBJ Sequence Read Archive (DRA) under the bioproject ID PRJDB4176.

**Healthy Samples:**

| Run | BioProject | BioSample | Experiment |
| --- | --- | --- | --- |
| DRR127546 | PRJDB4176 | SAMD00114792 | DRX120290 |
| DRR171724 | PRJDB4176 | SAMD00114718 | DRX162336 |
| DRR171783 | PRJDB4176 | SAMD00114796 | DRX162395 |
| DRR171629 | PRJDB4176 | SAMD00164939 | DRX162241 |
| DRR127524 | PRJDB4176 | SAMD00114770 | DRX120268 |
| DRR171741 | PRJDB4176 | SAMD00114741 | DRX162353 |
| DRR171786 | PRJDB4176 | SAMD00114800 | DRX162398 |
| DRR171640 | PRJDB4176 | SAMD00164950 | DRX162252 |
| DRR127634 | PRJDB4176 | SAMD00114880 | DRX120378 |
| DRR171777 | PRJDB4176 | SAMD00114789 | DRX162389 |
| DRR171467 | PRJDB4176 | SAMD00164727 | DRX162079 |
| DRR171733 | PRJDB4176 | SAMD00114730 | DRX162345 |
| DRR171628 | PRJDB4176 | SAMD00164938 | DRX162240 |
| DRR127731 | PRJDB4176 | SAMD00114977 | DRX120475 |
| DRR171727 | PRJDB4176 | SAMD00114721 | DRX162339 |
| DRR171953 | PRJDB4176 | SAMD00115018 | DRX162565 |
| DRR171755 | PRJDB4176 | SAMD00114757 | DRX162367 |
| DRR171964 | PRJDB4176 | SAMD00115032 | DRX162576 |
| DRR127707 | PRJDB4176 | SAMD00114953 | DRX120451 |
| DRR171581 | PRJDB4176 | SAMD00164891 | DRX162193 |
| DRR171617 | PRJDB4176 | SAMD00164927 | DRX162229 |
| DRR171954 | PRJDB4176 | SAMD00115019 | DRX162566 |
| DRR171653 | PRJDB4176 | SAMD00164963 | DRX162265 |
| DRR171645 | PRJDB4176 | SAMD00164955 | DRX162257 |
| DRR171779 | PRJDB4176 | SAMD00114791 | DRX162391 |
| DRR127724 | PRJDB4176 | SAMD00114970 | DRX120468 |
| DRR171959 | PRJDB4176 | SAMD00115027 | DRX162571 |
| DRR171639 | PRJDB4176 | SAMD00164949 | DRX162251 |
| DRR171747 | PRJDB4176 | SAMD00114747 | DRX162359 |
| DRR171637 | PRJDB4176 | SAMD00164947 | DRX162249 |
| DRR171762 | PRJDB4176 | SAMD00114769 | DRX162374 |
| DRR171500 | PRJDB4176 | SAMD00164806 | DRX162112 |
| DRR171503 | PRJDB4176 | SAMD00164809 | DRX162115 |
| DRR127751 | PRJDB4176 | SAMD00114997 | DRX120495 |
| DRR171499 | PRJDB4176 | SAMD00164805 | DRX162111 |
| DRR127776 | PRJDB4176 | SAMD00115022 | DRX120520 |
| DRR171642 | PRJDB4176 | SAMD00164952 | DRX162254 |
| DRR171588 | PRJDB4176 | SAMD00164898 | DRX162200 |
| DRR171770 | PRJDB4176 | SAMD00114779 | DRX162382 |
| DRR171694 | PRJDB4176 | SAMD00165004 | DRX162306 |
| DRR171546 | PRJDB4176 | SAMD00164856 | DRX162158 |
| DRR171610 | PRJDB4176 | SAMD00164920 | DRX162222 |
| DRR127777 | PRJDB4176 | SAMD00115023 | DRX120521 |
| DRR171619 | PRJDB4176 | SAMD00164929 | DRX162231 |
| DRR127728 | PRJDB4176 | SAMD00114974 | DRX120472 |
| DRR171673 | PRJDB4176 | SAMD00164983 | DRX162285 |
| DRR171659 | PRJDB4176 | SAMD00164969 | DRX162271 |
| DRR171698 | PRJDB4176 | SAMD00165008 | DRX162310 |
| DRR171616 | PRJDB4176 | SAMD00164926 | DRX162228 |
| DRR127762 | PRJDB4176 | SAMD00115008 | DRX120506 |
| DRR171691 | PRJDB4176 | SAMD00165001 | DRX162303 |
| DRR127756 | PRJDB4176 | SAMD00115002 | DRX120500 |
| DRR171497 | PRJDB4176 | SAMD00164803 | DRX162109 |
| DRR127583 | PRJDB4176 | SAMD00114829 | DRX120327 |
| DRR171594 | PRJDB4176 | SAMD00164904 | DRX162206 |
| DRR171613 | PRJDB4176 | SAMD00164923 | DRX162225 |
| DRR171620 | PRJDB4176 | SAMD00164930 | DRX162232 |
| DRR171686 | PRJDB4176 | SAMD00164996 | DRX162298 |
| DRR171782 | PRJDB4176 | SAMD00114795 | DRX162394 |
| DRR127713 | PRJDB4176 | SAMD00114959 | DRX120457 |
| DRR171601 | PRJDB4176 | SAMD00164911 | DRX162213 |
| DRR171631 | PRJDB4176 | SAMD00164941 | DRX162243 |
| DRR171663 | PRJDB4176 | SAMD00164973 | DRX162275 |
| DRR127752 | PRJDB4176 | SAMD00114998 | DRX120496 |
| DRR171606 | PRJDB4176 | SAMD00164916 | DRX162218 |
| DRR171676 | PRJDB4176 | SAMD00164986 | DRX162288 |
| DRR171965 | PRJDB4176 | SAMD00115033 | DRX162577 |
| DRR171469 | PRJDB4176 | SAMD00164729 | DRX162081 |
| DRR171791 | PRJDB4176 | SAMD00114807 | DRX162403 |
| DRR171710 | PRJDB4176 | SAMD00165020 | DRX162322 |
| DRR127763 | PRJDB4176 | SAMD00115009 | DRX120507 |
| DRR171538 | PRJDB4176 | SAMD00164848 | DRX162150 |
| DRR171641 | PRJDB4176 | SAMD00164951 | DRX162253 |
| DRR171725 | PRJDB4176 | SAMD00114719 | DRX162337 |
| DRR171608 | PRJDB4176 | SAMD00164918 | DRX162220 |
| DRR171580 | PRJDB4176 | SAMD00164890 | DRX162192 |
| DRR171651 | PRJDB4176 | SAMD00164961 | DRX162263 |
| DRR171607 | PRJDB4176 | SAMD00164917 | DRX162219 |
| DRR171957 | PRJDB4176 | SAMD00115025 | DRX162569 |
| DRR171646 | PRJDB4176 | SAMD00164956 | DRX162258 |
| DRR171655 | PRJDB4176 | SAMD00164965 | DRX162267 |
| DRR171644 | PRJDB4176 | SAMD00164954 | DRX162256 |
| DRR171807 | PRJDB4176 | SAMD00114824 | DRX162419 |
| DRR171648 | PRJDB4176 | SAMD00164958 | DRX162260 |
| DRR171652 | PRJDB4176 | SAMD00164962 | DRX162264 |
| DRR171509 | PRJDB4176 | SAMD00164819 | DRX162121 |
| DRR127616 | PRJDB4176 | SAMD00114862 | DRX120360 |
| DRR171810 | PRJDB4176 | SAMD00114830 | DRX162422 |
| DRR171572 | PRJDB4176 | SAMD00164882 | DRX162184 |
| DRR171621 | PRJDB4176 | SAMD00164931 | DRX162233 |
| DRR171793 | PRJDB4176 | SAMD00114809 | DRX162405 |
| DRR171657 | PRJDB4176 | SAMD00164967 | DRX162269 |
| DRR127721 | PRJDB4176 | SAMD00114967 | DRX120465 |
| DRR171643 | PRJDB4176 | SAMD00164953 | DRX162255 |
| DRR171506 | PRJDB4176 | SAMD00164814 | DRX162118 |
| DRR127755 | PRJDB4176 | SAMD00115001 | DRX120499 |
| DRR171488 | PRJDB4176 | SAMD00164774 | DRX162100 |
| DRR171773 | PRJDB4176 | SAMD00114785 | DRX162385 |
| DRR127683 | PRJDB4176 | SAMD00114929 | DRX120427 |
| DRR171598 | PRJDB4176 | SAMD00164908 | DRX162210 |
| DRR171591 | PRJDB4176 | SAMD00164901 | DRX162203 |
| DRR171796 | PRJDB4176 | SAMD00114812 | DRX162408 |
| DRR171737 | PRJDB4176 | SAMD00114736 | DRX162349 |
| DRR171477 | PRJDB4176 | SAMD00164756 | DRX162089 |
| DRR127672 | PRJDB4176 | SAMD00114918 | DRX120416 |
| DRR171604 | PRJDB4176 | SAMD00164914 | DRX162216 |
| DRR127704 | PRJDB4176 | SAMD00114950 | DRX120448 |
| DRR127628 | PRJDB4176 | SAMD00114874 | DRX120372 |
| DRR171812 | PRJDB4176 | SAMD00114832 | DRX162424 |
| DRR171578 | PRJDB4176 | SAMD00164888 | DRX162190 |
| DRR171711 | PRJDB4176 | SAMD00165021 | DRX162323 |
| DRR162775 | PRJDB4176 | SAMD00154991 | DRX153394 |
| DRR171567 | PRJDB4176 | SAMD00164877 | DRX162179 |
| DRR127736 | PRJDB4176 | SAMD00114982 | DRX120480 |
| DRR171662 | PRJDB4176 | SAMD00164972 | DRX162274 |
| DRR127613 | PRJDB4176 | SAMD00114859 | DRX120357 |
| DRR127537 | PRJDB4176 | SAMD00114783 | DRX120281 |
| DRR127649 | PRJDB4176 | SAMD00114895 | DRX120393 |
| DRR127588 | PRJDB4176 | SAMD00114834 | DRX120332 |
| DRR171592 | PRJDB4176 | SAMD00164902 | DRX162204 |
| DRR171552 | PRJDB4176 | SAMD00164862 | DRX162164 |
| DRR171813 | PRJDB4176 | SAMD00114833 | DRX162425 |
| DRR171599 | PRJDB4176 | SAMD00164909 | DRX162211 |
| DRR171515 | PRJDB4176 | SAMD00164825 | DRX162127 |
| DRR171605 | PRJDB4176 | SAMD00164915 | DRX162217 |
| DRR171656 | PRJDB4176 | SAMD00164966 | DRX162268 |
| DRR171765 | PRJDB4176 | SAMD00114773 | DRX162377 |
| DRR171585 | PRJDB4176 | SAMD00164895 | DRX162197 |
| DRR171726 | PRJDB4176 | SAMD00114720 | DRX162338 |
| DRR171700 | PRJDB4176 | SAMD00165010 | DRX162312 |
| DRR127596 | PRJDB4176 | SAMD00114842 | DRX120340 |
| DRR171801 | PRJDB4176 | SAMD00114818 | DRX162413 |
| DRR171527 | PRJDB4176 | SAMD00164837 | DRX162139 |
| DRR127692 | PRJDB4176 | SAMD00114938 | DRX120436 |
| DRR171555 | PRJDB4176 | SAMD00164865 | DRX162167 |
| DRR171587 | PRJDB4176 | SAMD00164897 | DRX162199 |
| DRR171798 | PRJDB4176 | SAMD00114815 | DRX162410 |
| DRR171816 | PRJDB4176 | SAMD00114837 | DRX162428 |
| DRR171654 | PRJDB4176 | SAMD00164964 | DRX162266 |
| DRR127748 | PRJDB4176 | SAMD00114994 | DRX120492 |
| DRR171474 | PRJDB4176 | SAMD00164748 | DRX162086 |
| DRR127532 | PRJDB4176 | SAMD00114778 | DRX120276 |
| DRR171563 | PRJDB4176 | SAMD00164873 | DRX162175 |
| DRR171763 | PRJDB4176 | SAMD00114771 | DRX162375 |
| DRR171586 | PRJDB4176 | SAMD00164896 | DRX162198 |
| DRR127619 | PRJDB4176 | SAMD00114865 | DRX120363 |
| DRR171487 | PRJDB4176 | SAMD00164773 | DRX162099 |
| DRR171817 | PRJDB4176 | SAMD00114838 | DRX162429 |
| DRR127597 | PRJDB4176 | SAMD00114843 | DRX120341 |
| DRR171569 | PRJDB4176 | SAMD00164879 | DRX162181 |
| DRR171650 | PRJDB4176 | SAMD00164960 | DRX162262 |
| DRR173016 | PRJDB4176 | SAMD00136648 | DRX163627 |
| DRR171571 | PRJDB4176 | SAMD00164881 | DRX162183 |
| DRR171802 | PRJDB4176 | SAMD00114819 | DRX162414 |
| DRR171479 | PRJDB4176 | SAMD00164758 | DRX162091 |
| DRR171772 | PRJDB4176 | SAMD00114784 | DRX162384 |
| DRR162776 | PRJDB4176 | SAMD00154992 | DRX153395 |
| DRR171514 | PRJDB4176 | SAMD00164824 | DRX162126 |
| DRR171705 | PRJDB4176 | SAMD00165015 | DRX162317 |
| DRR171689 | PRJDB4176 | SAMD00164999 | DRX162301 |
| DRR171513 | PRJDB4176 | SAMD00164823 | DRX162125 |
| DRR171745 | PRJDB4176 | SAMD00114745 | DRX162357 |
| DRR171771 | PRJDB4176 | SAMD00114780 | DRX162383 |
| DRR171539 | PRJDB4176 | SAMD00164849 | DRX162151 |
| DRR171525 | PRJDB4176 | SAMD00164835 | DRX162137 |
| DRR127535 | PRJDB4176 | SAMD00114781 | DRX120279 |
| DRR127552 | PRJDB4176 | SAMD00114798 | DRX120296 |
| DRR171523 | PRJDB4176 | SAMD00164833 | DRX162135 |
| DRR171568 | PRJDB4176 | SAMD00164878 | DRX162180 |
| DRR171530 | PRJDB4176 | SAMD00164840 | DRX162142 |
| DRR171543 | PRJDB4176 | SAMD00164853 | DRX162155 |
| DRR171517 | PRJDB4176 | SAMD00164827 | DRX162129 |
| DRR171545 | PRJDB4176 | SAMD00164855 | DRX162157 |
| DRR171647 | PRJDB4176 | SAMD00164957 | DRX162259 |
| DRR171589 | PRJDB4176 | SAMD00164899 | DRX162201 |
| DRR171518 | PRJDB4176 | SAMD00164828 | DRX162130 |
| DRR171560 | PRJDB4176 | SAMD00164870 | DRX162172 |
| DRR171576 | PRJDB4176 | SAMD00164886 | DRX162188 |

**CRC Samples:**

| **Run** | **BioProject** | **BioSample** | **Experiment** |
| --- | --- | --- | --- |
| DRR171910 | PRJDB4176 | SAMD00114957 | DRX162522 |
| DRR171750 | PRJDB4176 | SAMD00114750 | DRX162362 |
| DRR171784 | PRJDB4176 | SAMD00114797 | DRX162396 |
| DRR127515 | PRJDB4176 | SAMD00114761 | DRX120259 |
| DRR171702 | PRJDB4176 | SAMD00165012 | DRX162314 |
| DRR127485 | PRJDB4176 | SAMD00114731 | DRX120229 |
| DRR171871 | PRJDB4176 | SAMD00114907 | DRX162483 |
| DRR127481 | PRJDB4176 | SAMD00114727 | DRX120225 |
| DRR127519 | PRJDB4176 | SAMD00114765 | DRX120263 |
| DRR127476 | PRJDB4176 | SAMD00114722 | DRX120220 |
| DRR171767 | PRJDB4176 | SAMD00114775 | DRX162379 |
| DRR162778 | PRJDB4176 | SAMD00154994 | DRX153397 |
| DRR127517 | PRJDB4176 | SAMD00114763 | DRX120261 |
| DRR171697 | PRJDB4176 | SAMD00165007 | DRX162309 |
| DRR171742 | PRJDB4176 | SAMD00114742 | DRX162354 |
| DRR127521 | PRJDB4176 | SAMD00114767 | DRX120265 |
| DRR127720 | PRJDB4176 | SAMD00114966 | DRX120464 |
| DRR171704 | PRJDB4176 | SAMD00165014 | DRX162316 |
| DRR171661 | PRJDB4176 | SAMD00164971 | DRX162273 |
| DRR171794 | PRJDB4176 | SAMD00114810 | DRX162406 |
| DRR127536 | PRJDB4176 | SAMD00114782 | DRX120280 |
| DRR127789 | PRJDB4176 | SAMD00115035 | DRX120533 |
| DRR127674 | PRJDB4176 | SAMD00114920 | DRX120418 |
| DRR127767 | PRJDB4176 | SAMD00115013 | DRX120511 |
| DRR127754 | PRJDB4176 | SAMD00115000 | DRX120498 |
| DRR171805 | PRJDB4176 | SAMD00114822 | DRX162417 |
| DRR127559 | PRJDB4176 | SAMD00114805 | DRX120303 |
| DRR171667 | PRJDB4176 | SAMD00164977 | DRX162279 |
| DRR127478 | PRJDB4176 | SAMD00114724 | DRX120222 |
| DRR127580 | PRJDB4176 | SAMD00114826 | DRX120324 |
| DRR171672 | PRJDB4176 | SAMD00164982 | DRX162284 |
| DRR171665 | PRJDB4176 | SAMD00164975 | DRX162277 |
| DRR127509 | PRJDB4176 | SAMD00114755 | DRX120253 |
| DRR127579 | PRJDB4176 | SAMD00114825 | DRX120323 |
| DRR127625 | PRJDB4176 | SAMD00114871 | DRX120369 |
| DRR127685 | PRJDB4176 | SAMD00114931 | DRX120429 |
| DRR171685 | PRJDB4176 | SAMD00164995 | DRX162297 |
| DRR171858 | PRJDB4176 | SAMD00114891 | DRX162470 |
| DRR127666 | PRJDB4176 | SAMD00114912 | DRX120410 |
| DRR127646 | PRJDB4176 | SAMD00114892 | DRX120390 |
| DRR171683 | PRJDB4176 | SAMD00164993 | DRX162295 |
| DRR171693 | PRJDB4176 | SAMD00165003 | DRX162305 |
| DRR171764 | PRJDB4176 | SAMD00114772 | DRX162376 |
| DRR171472 | PRJDB4176 | SAMD00164745 | DRX162084 |
| DRR127567 | PRJDB4176 | SAMD00114813 | DRX120311 |
| DRR171859 | PRJDB4176 | SAMD00114894 | DRX162471 |
| DRR171891 | PRJDB4176 | SAMD00114933 | DRX162503 |
| DRR171666 | PRJDB4176 | SAMD00164976 | DRX162278 |
| DRR171905 | PRJDB4176 | SAMD00114949 | DRX162517 |
| DRR127774 | PRJDB4176 | SAMD00115020 | DRX120518 |
| DRR171519 | PRJDB4176 | SAMD00164829 | DRX162131 |
| DRR127718 | PRJDB4176 | SAMD00114964 | DRX120462 |
| DRR171909 | PRJDB4176 | SAMD00114956 | DRX162521 |
| DRR127594 | PRJDB4176 | SAMD00114840 | DRX120338 |
| DRR127647 | PRJDB4176 | SAMD00114893 | DRX120391 |
| DRR171800 | PRJDB4176 | SAMD00114817 | DRX162412 |
| DRR171819 | PRJDB4176 | SAMD00114841 | DRX162431 |
| DRR127507 | PRJDB4176 | SAMD00114753 | DRX120251 |
| DRR162777 | PRJDB4176 | SAMD00154993 | DRX153396 |
| DRR171459 | PRJDB4176 | SAMD00164694 | DRX162071 |
| DRR171688 | PRJDB4176 | SAMD00164998 | DRX162300 |
| DRR171684 | PRJDB4176 | SAMD00164994 | DRX162296 |
| DRR127617 | PRJDB4176 | SAMD00114863 | DRX120361 |
| DRR127700 | PRJDB4176 | SAMD00114946 | DRX120444 |
| DRR127557 | PRJDB4176 | SAMD00114803 | DRX120301 |
| DRR127638 | PRJDB4176 | SAMD00114884 | DRX120382 |
| DRR127603 | PRJDB4176 | SAMD00114849 | DRX120347 |
| DRR127708 | PRJDB4176 | SAMD00114954 | DRX120452 |
| DRR127669 | PRJDB4176 | SAMD00114915 | DRX120413 |
| DRR162779 | PRJDB4176 | SAMD00154995 | DRX153398 |
| DRR171680 | PRJDB4176 | SAMD00164990 | DRX162292 |
| DRR127491 | PRJDB4176 | SAMD00114737 | DRX120235 |
| DRR127514 | PRJDB4176 | SAMD00114760 | DRX120258 |
| DRR171879 | PRJDB4176 | SAMD00114917 | DRX162491 |

**Supplemental Tables and Figures**

**
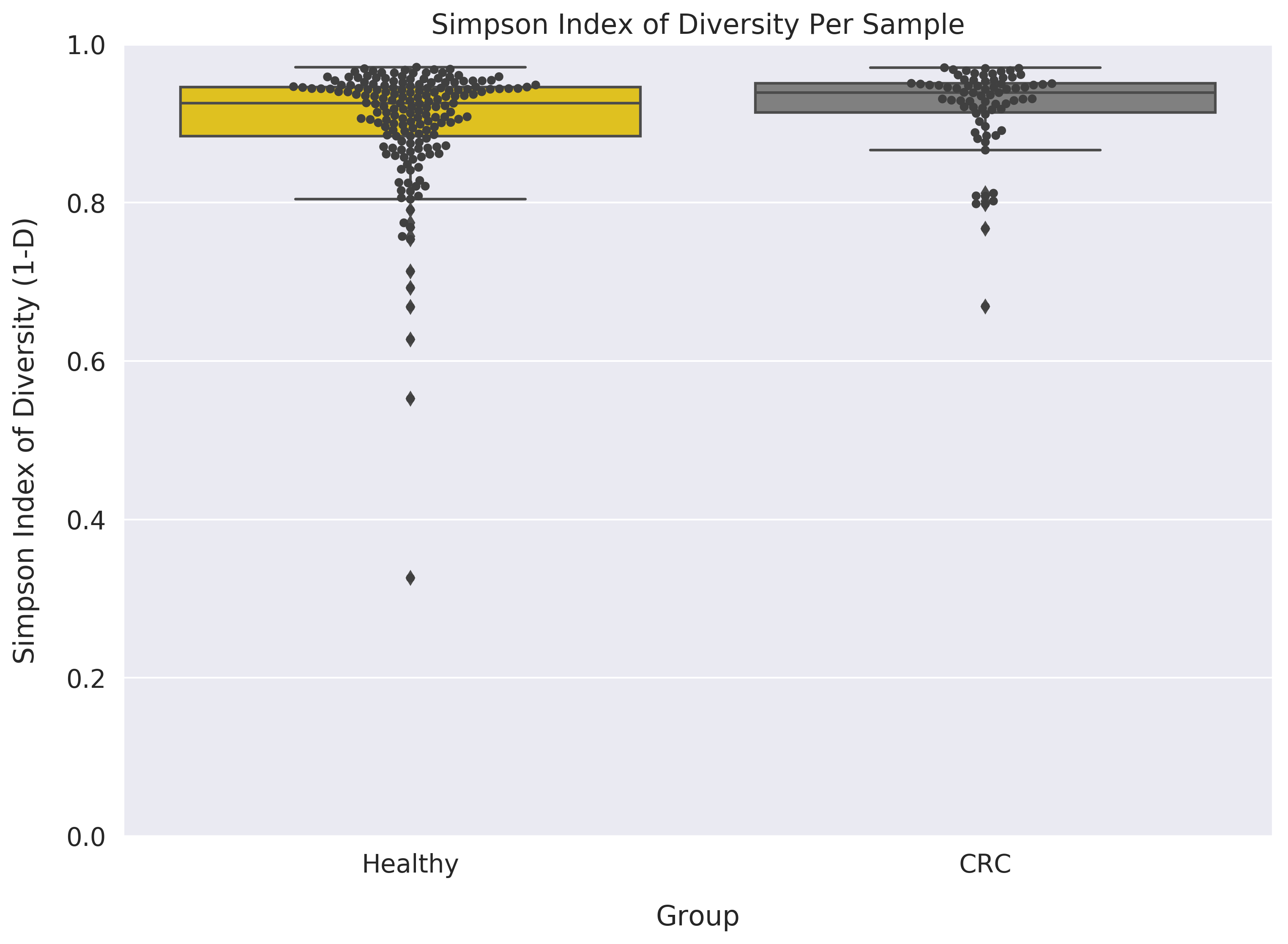
**

**Supplemental 1: Simpson index of Diversity within Healthy and CRC samples**

Boxplots displaying the Simpson index of Diversity (1-Dominance) for each sample from the Healthy and CRC sample groups. CRC samples were shown to contain a statistically significant higher species diversity (Mann-Whitney U test pvalue=0.023878) compared to those within Healthy samples.

**
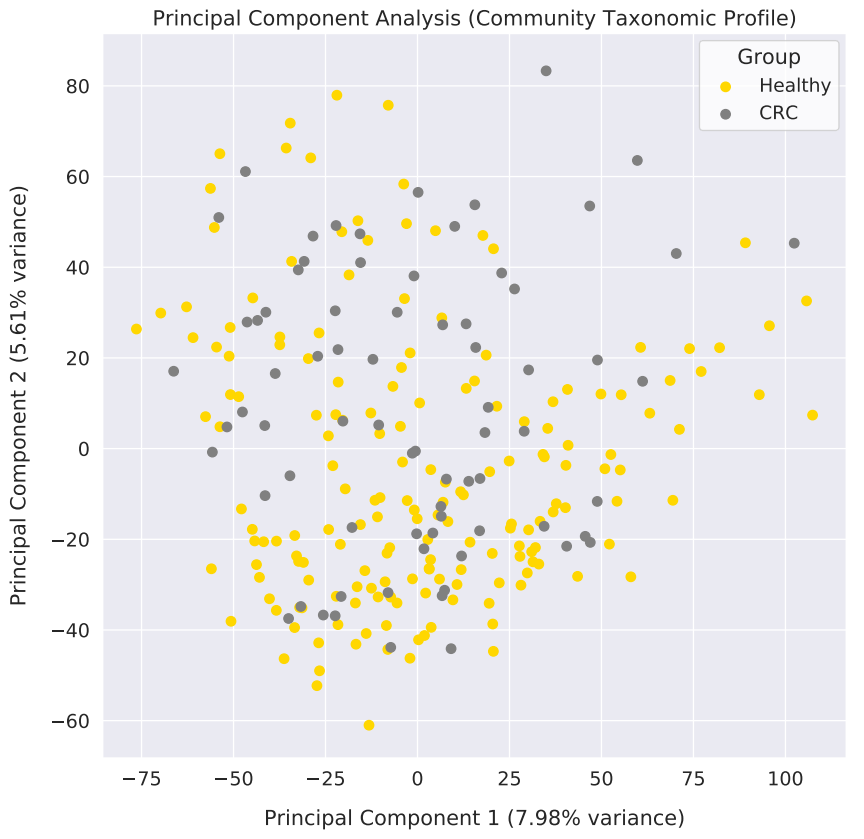
**

**Supplemental 2: PCA of Healthy and CRC Sample Taxonomic Profiles**

Principal components analysis of CLR-transformed bacterial species relative abundance sample profiles. Gold and grey circles represent Healthy and CRC samples, respectively. PCA exhibited little variance between Healthy and CRC-associated gut microbiome sample species relative abundance profiles.


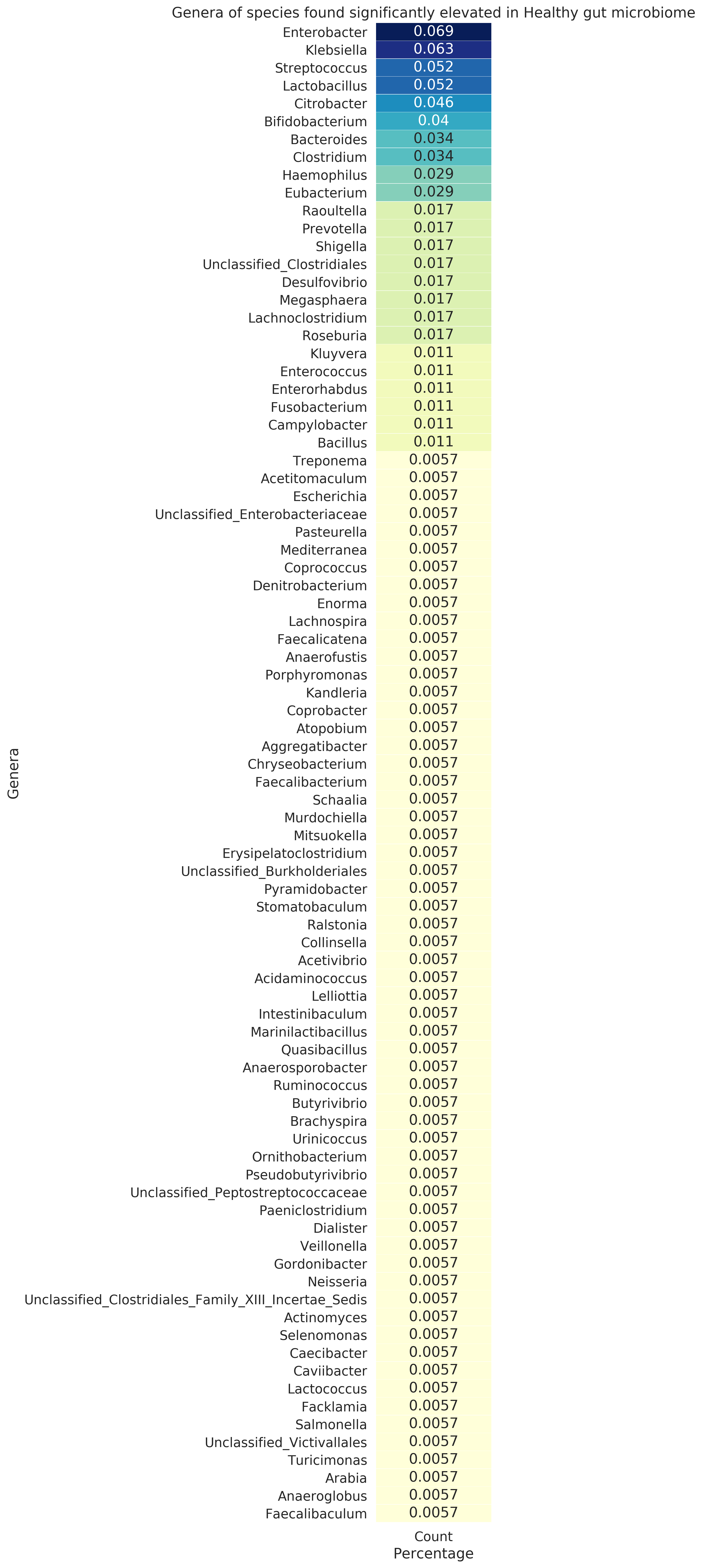


**Supplemental 3: Genera of 174 Species Reduced in Relative Abundance in CRC**

Heatmap showing the genera proportion of 174 species found in reduced relative abundance within the CRC gut microbiome compared

**a**


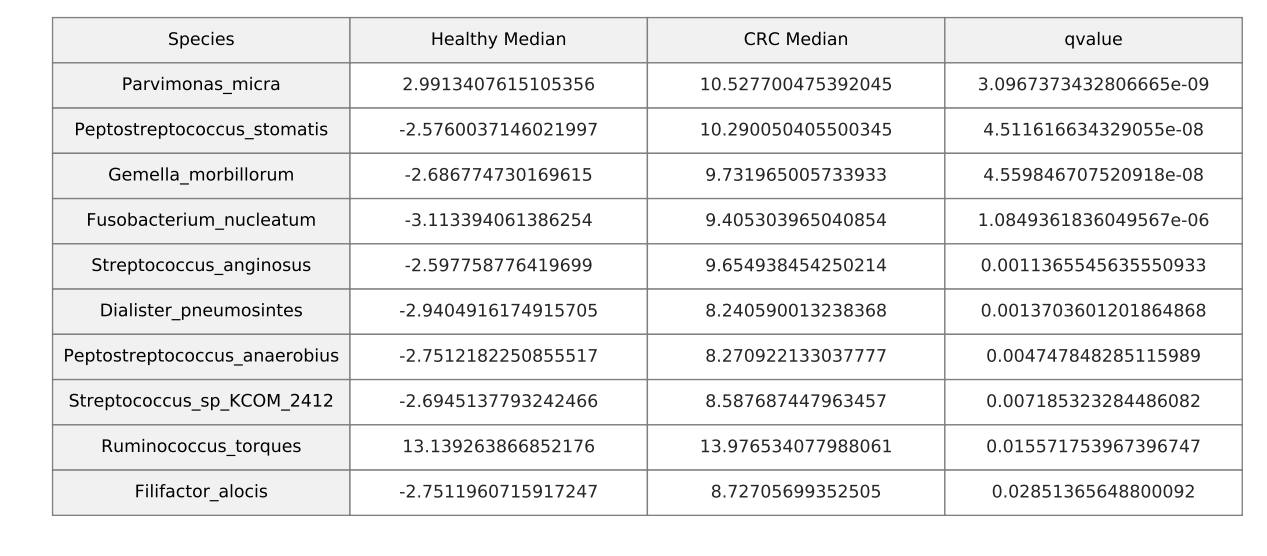
**b**

**
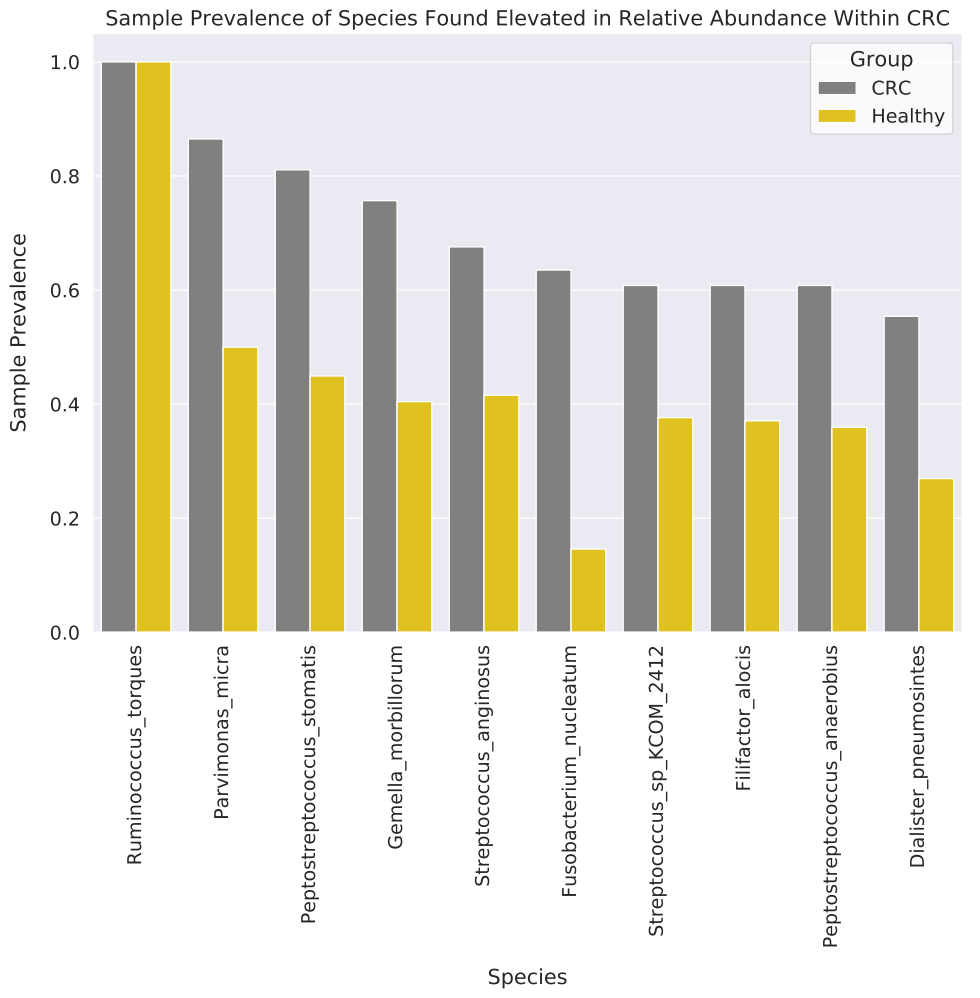
**

**c**

**
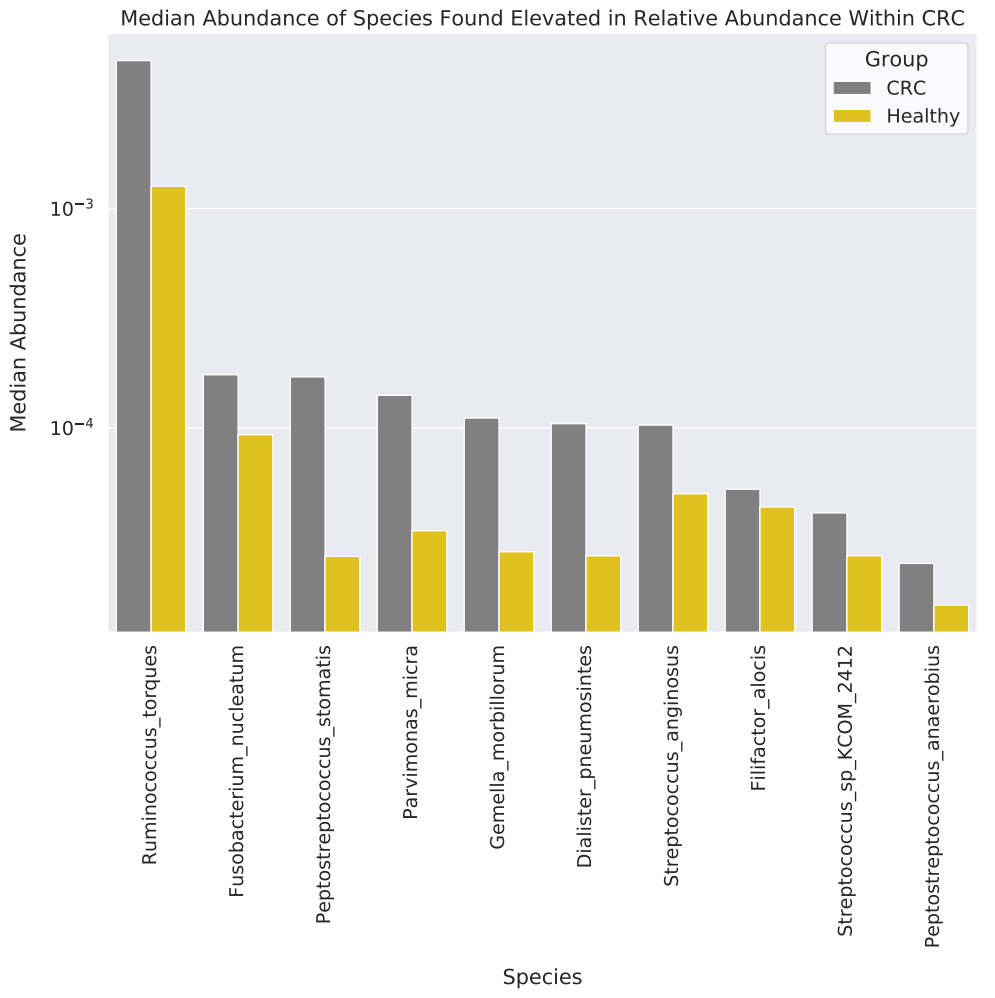
**

**Supplemental 4: Species Exhibiting Elevated Relative Abundance in CRC**

**a.** Table showing the 10 bacterial species found to be significantly (MWU-FDR qvalue<0.05) elevated in relative abundance within the CRC-associated gut microbiome compared to the Healthy gut microbiome. The median CLR-transformed relative abundance of species within Healthy and CRC samples is shown. **b.** Sample prevalence of the 10 species within the Healthy and CRC sample groups. **c.** Median relative abundance of the 10 species within the Healthy and CRC sample groups.

**
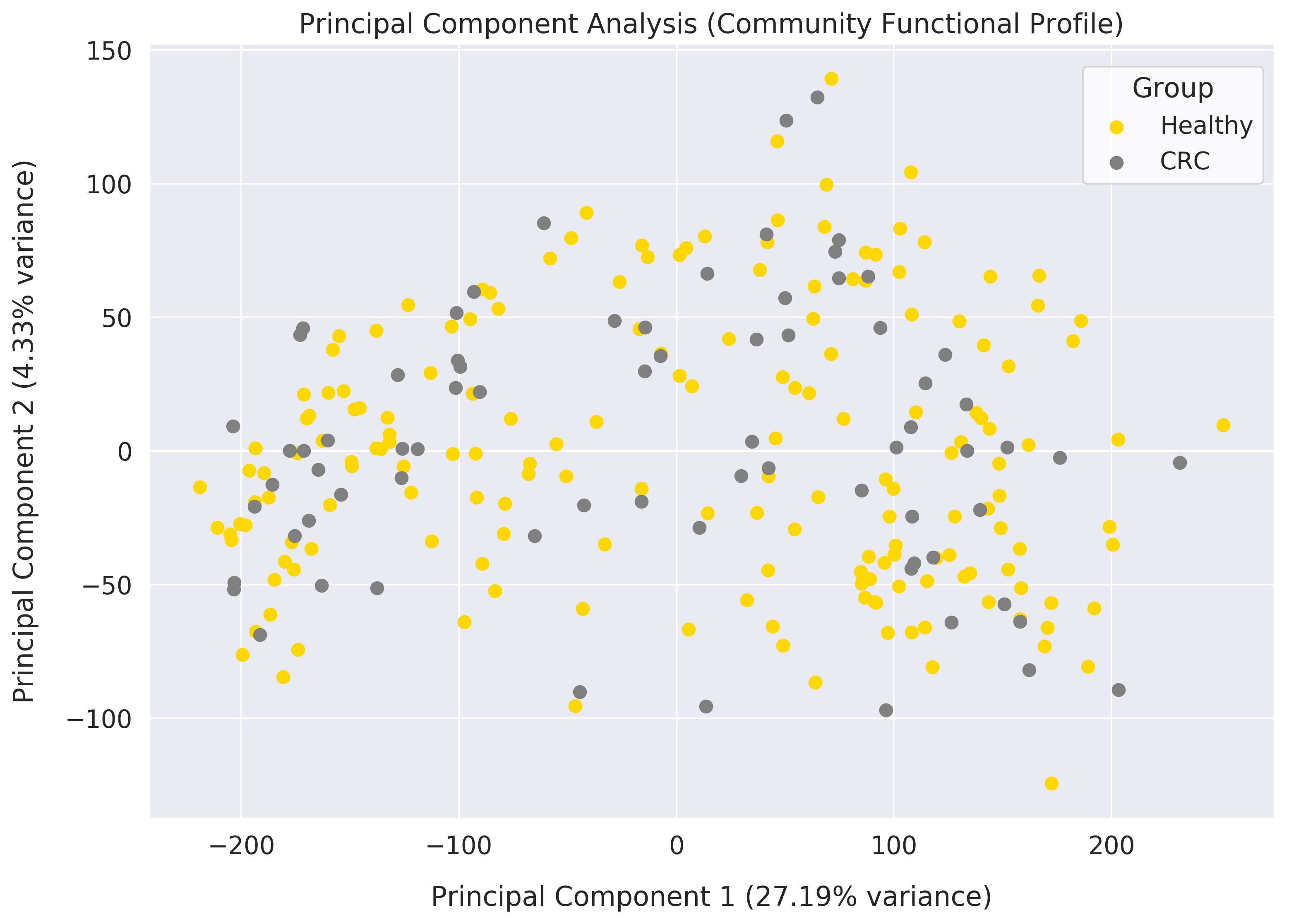
**

**Supplemental 5: Healthy and CRC Sample Functional Profile PCA**

Principal component analysis of CLR-transformed sample (Pfam) functional relative abundance profiles. Gold and grey dots represent Healthy or CRC samples, respectively.


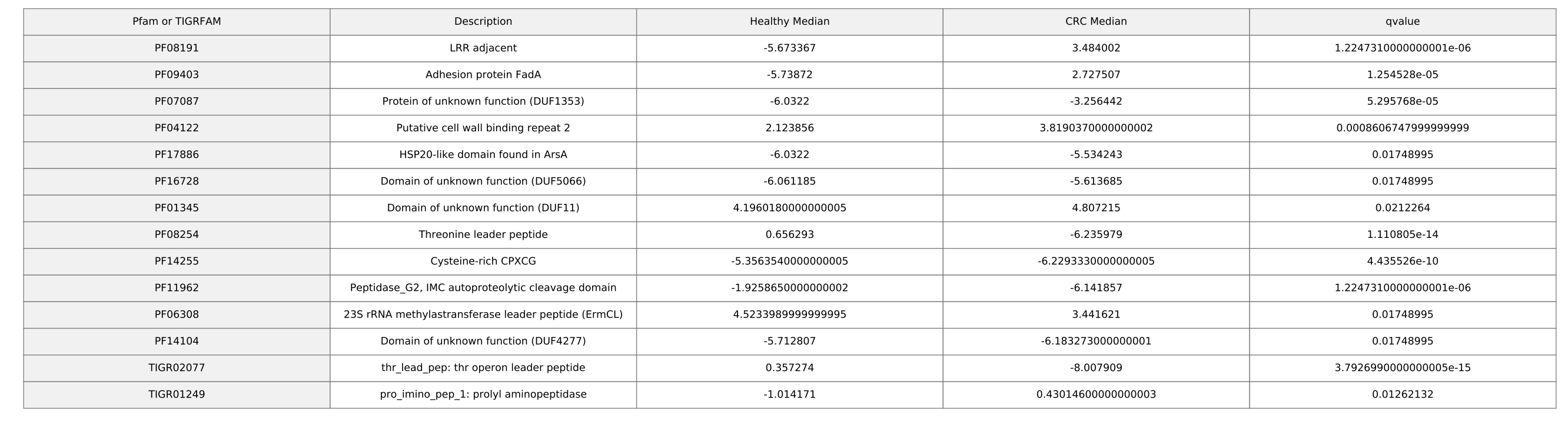


**Supplemental Table 1: Pfams and TIGRFAMs found in Differential Relative Abundance**

Table shows the 12 Pfams and 2 TIGRFAMs found to be differentially abundant between Healthy and CRC sample groups. Sample function relative abundances profiles were first CLR-transformed before testing for statistical significance (MWU-FDR: qvalue<0.05).

**a**


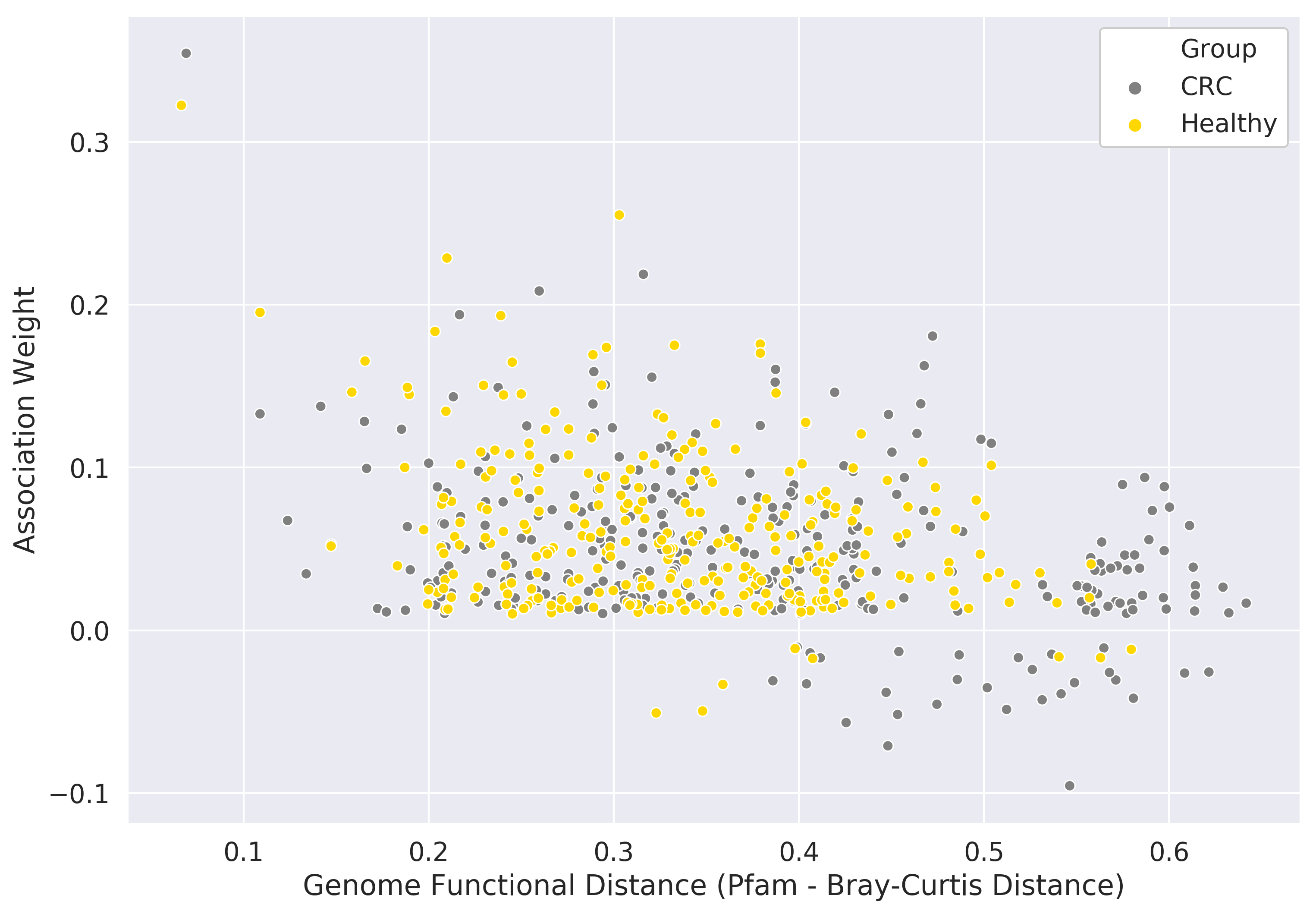


**b**

**
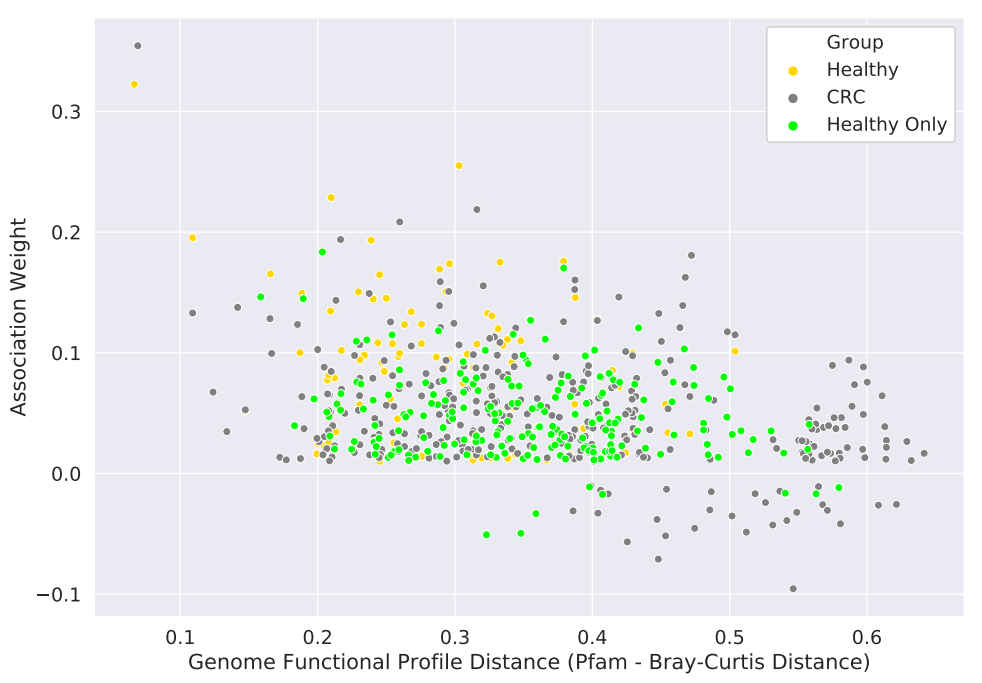
**

**c**


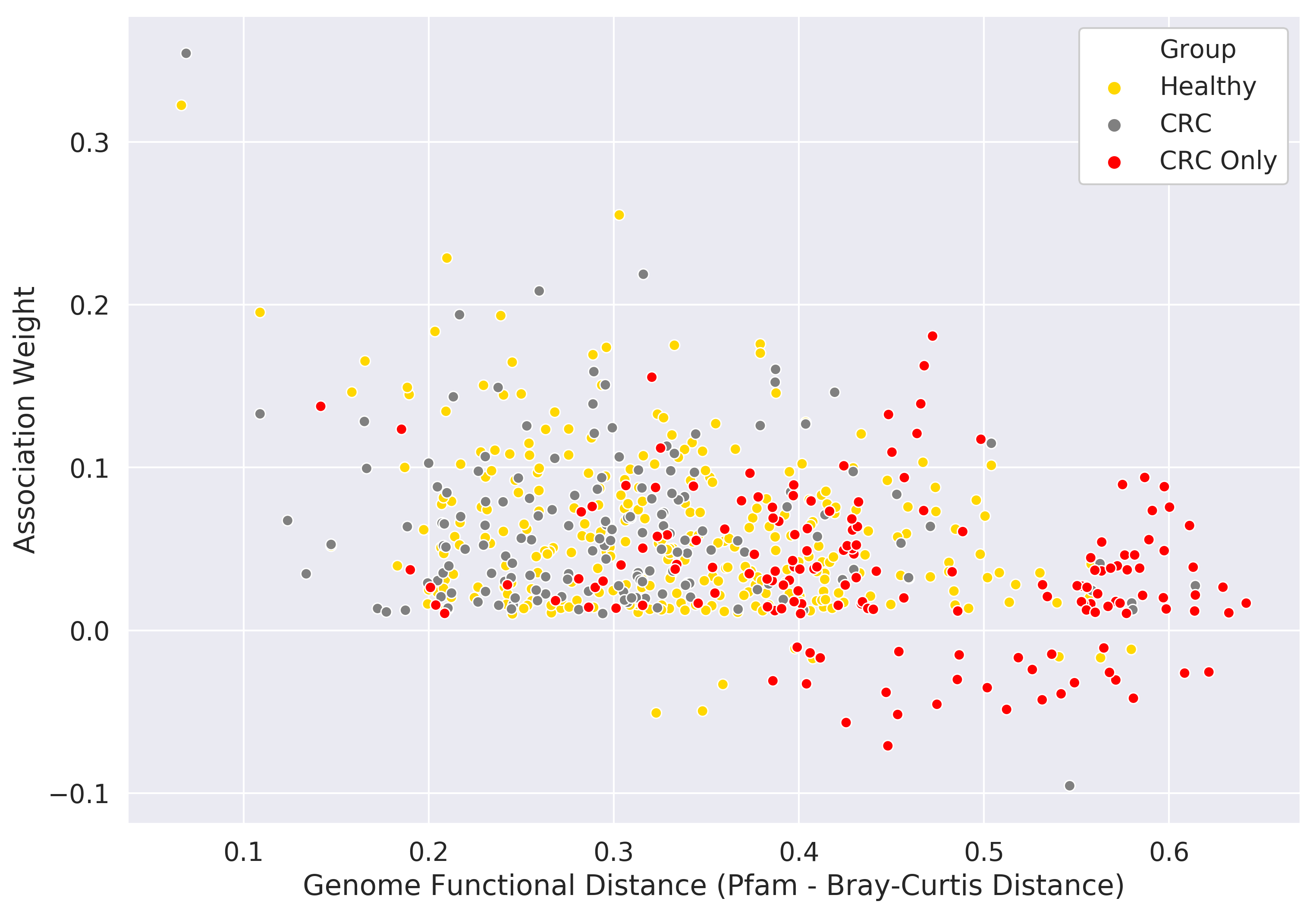


**Supplemental 6: Bacterial Partner Genome Functional Distance vs Association Weight**

**a,b,c.** Scatterplots showing bray-curtis distance between network partner bacterial genome functional (Pfam) profiles versus association weight. **a.** Displays all associations within Healthy and CRC networks. **b.** Highlights associations unique to the Healthy network in green. **c.** Highlights associations unique to the CRC network in red. The unique associations within the CRC network appear to occur between species with dissimilar genome functional profiles.

**a**


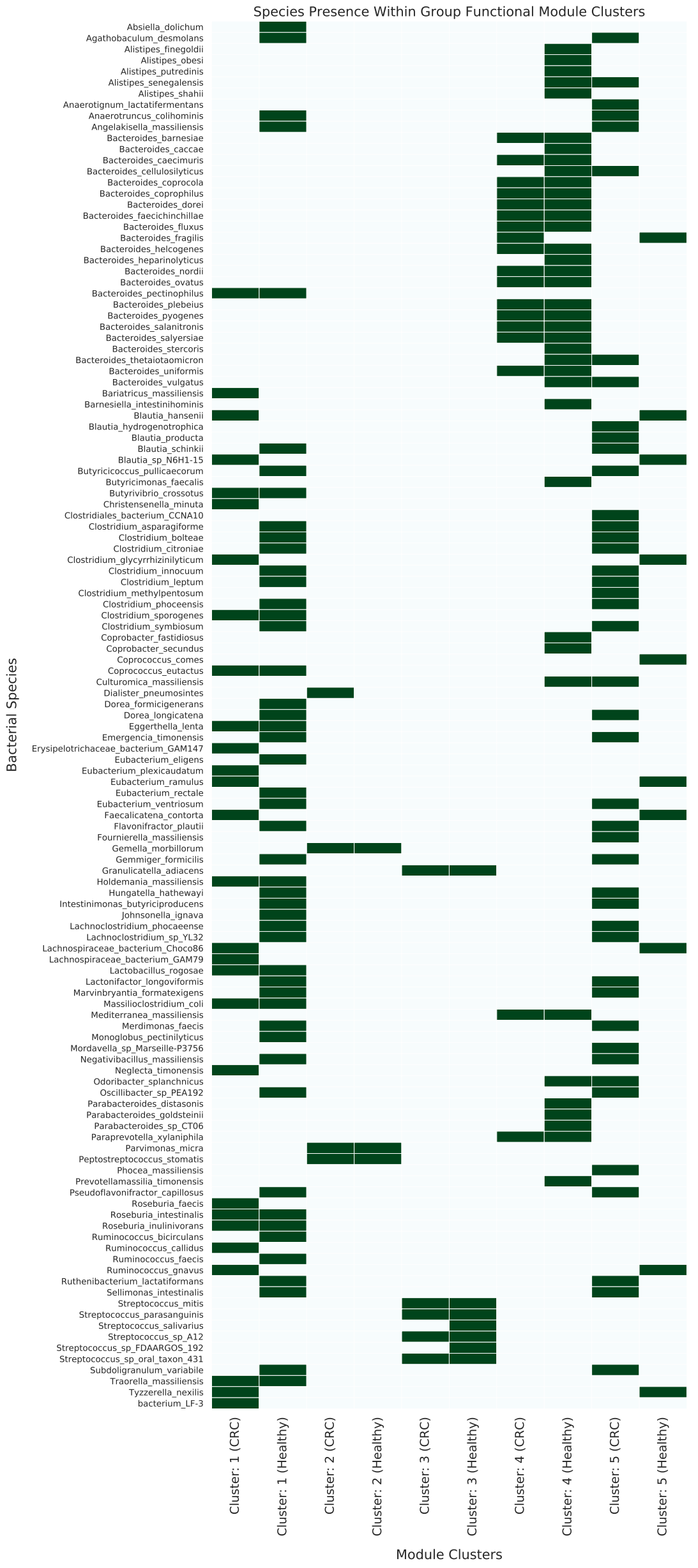


**b**

**
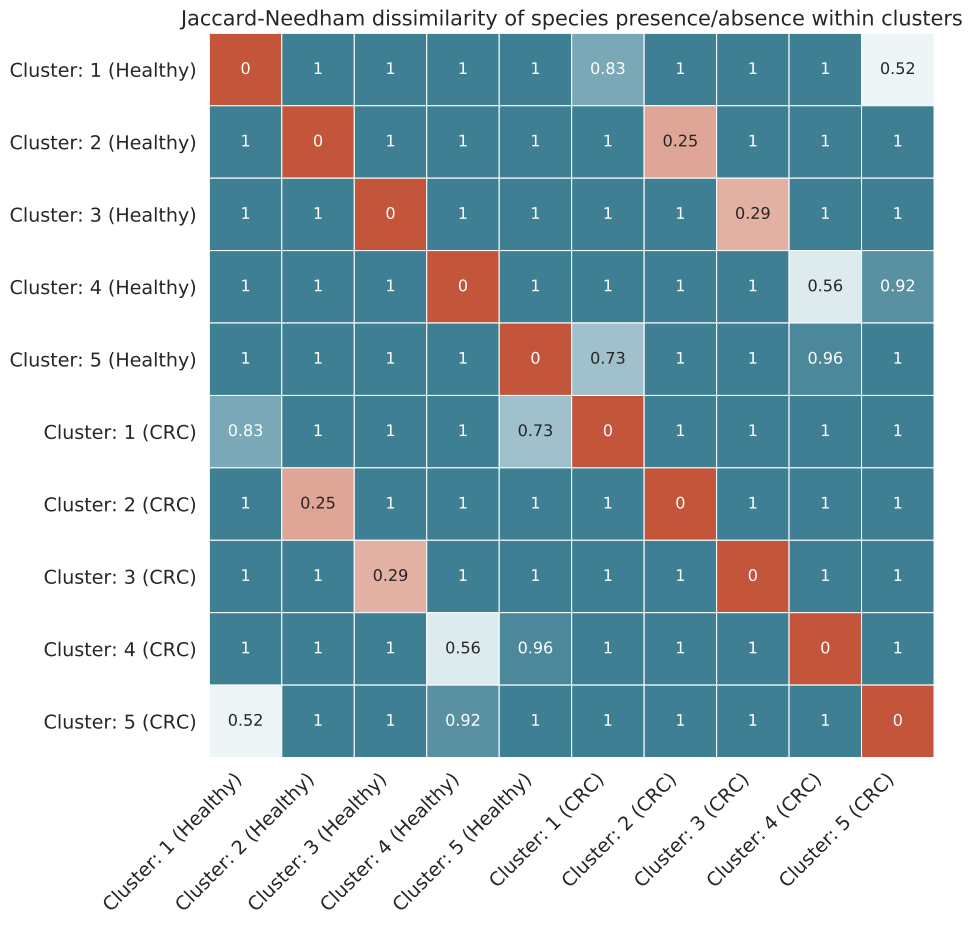
**

**
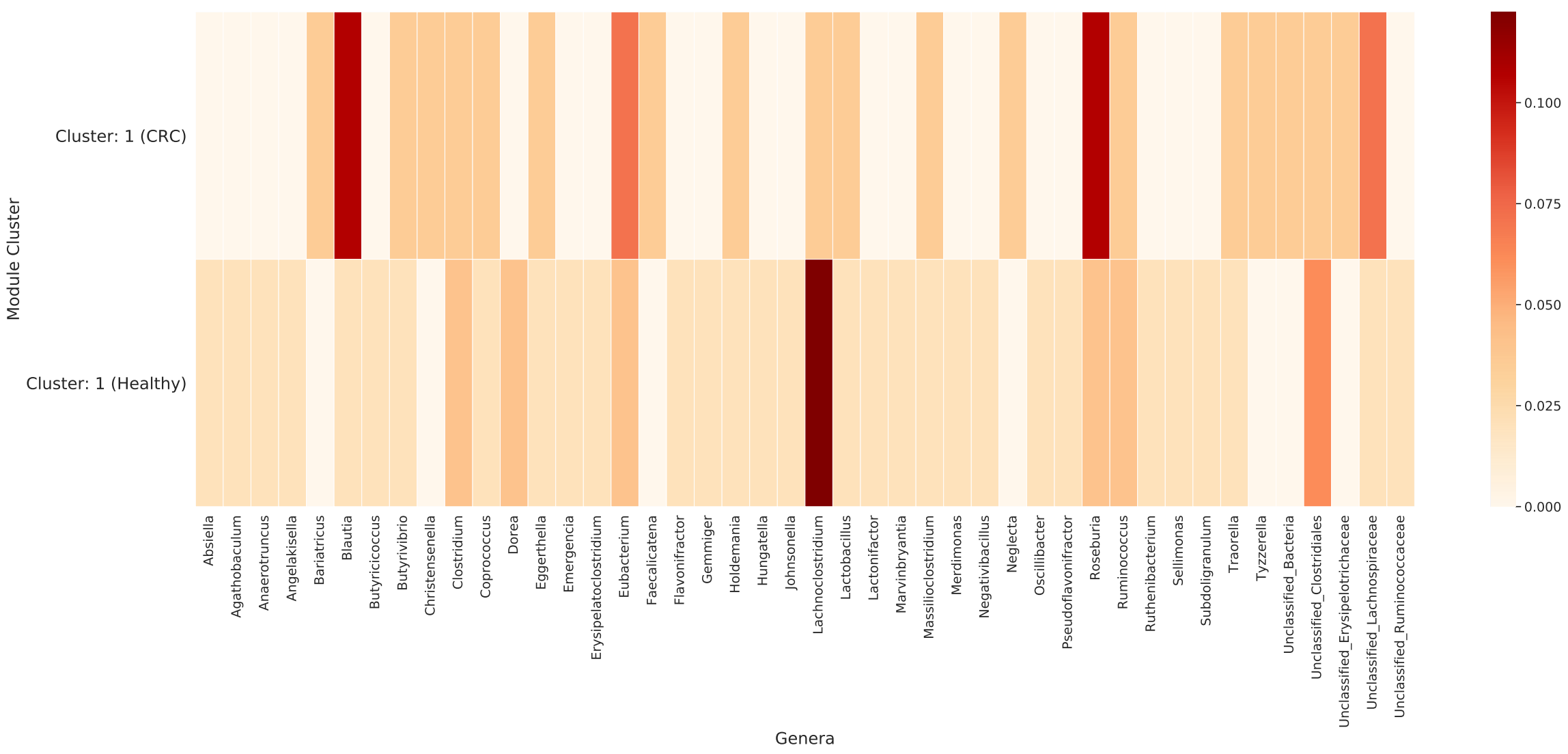
**

**c**

**d**

**
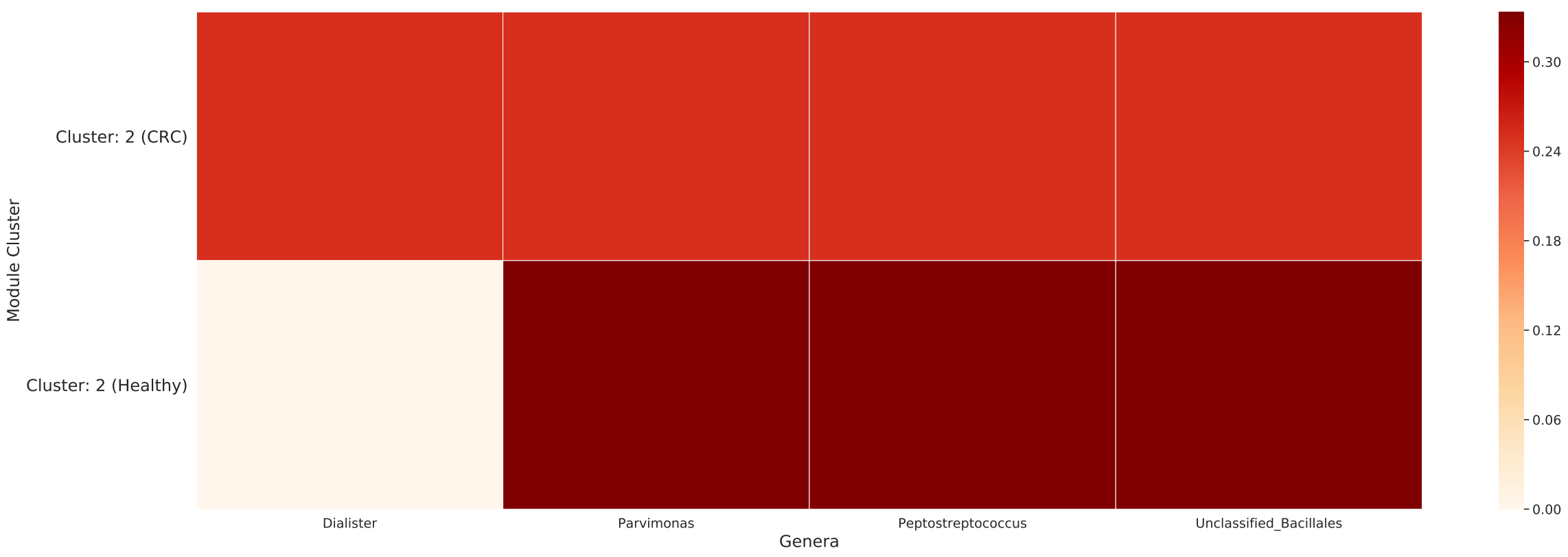
**

**e**

**
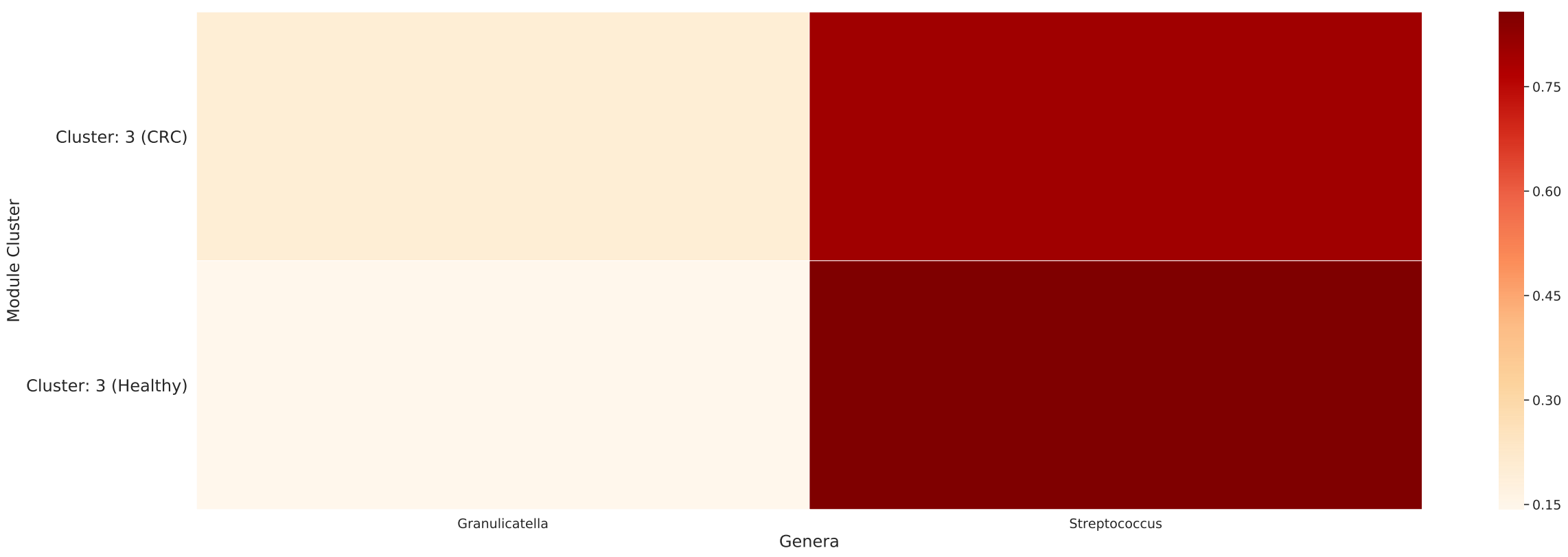
**

**f**

**
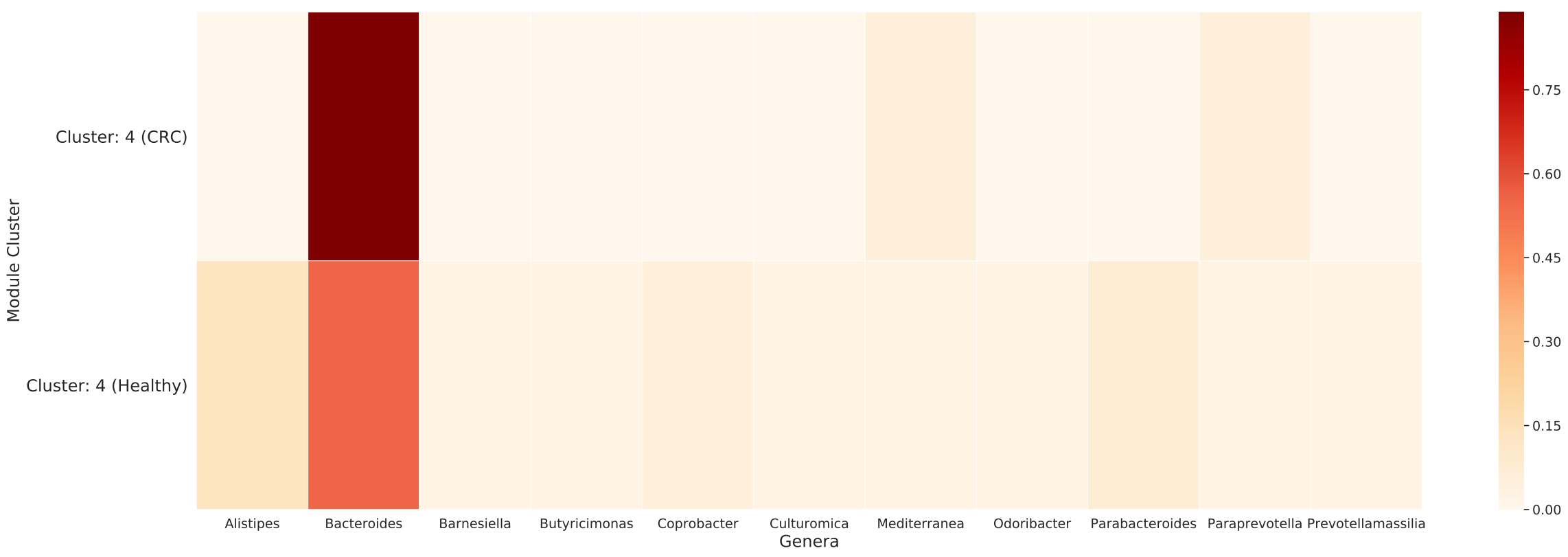
**

**g**

**
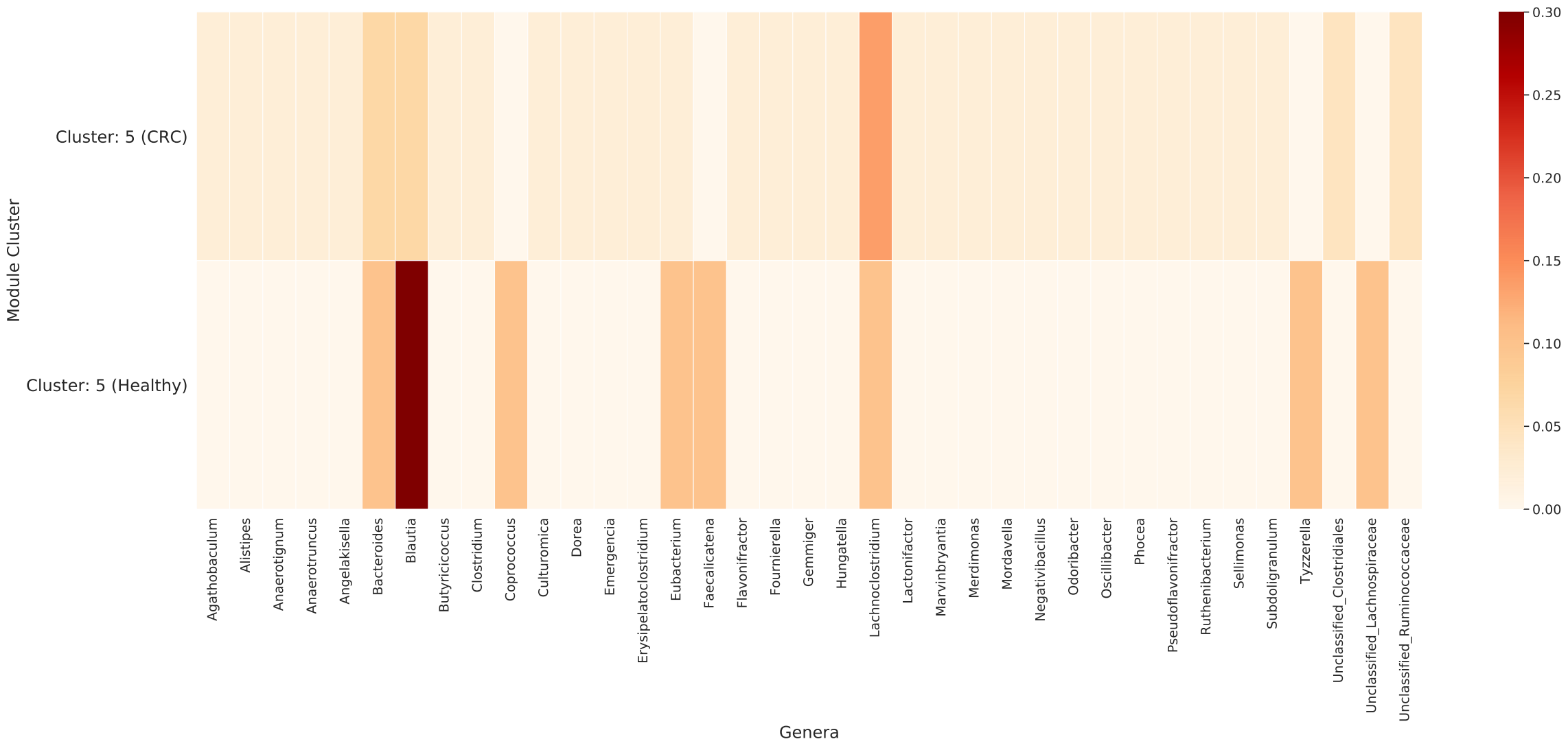
**

**Supplemental 7: Taxonomic Profiling of Module Clusters Within Networks**

**a.** Heatmap showing presence (dark green) and absence (light green) of species within module clusters of both group networks. **b**. Jaccard dissimilarity of group module cluster presence/absence profiles. **c,d,e,f,g**. Heatmaps showing genera proportion of species found within module cluster 1,2,3,4 or 5, respectively.

**a**

**
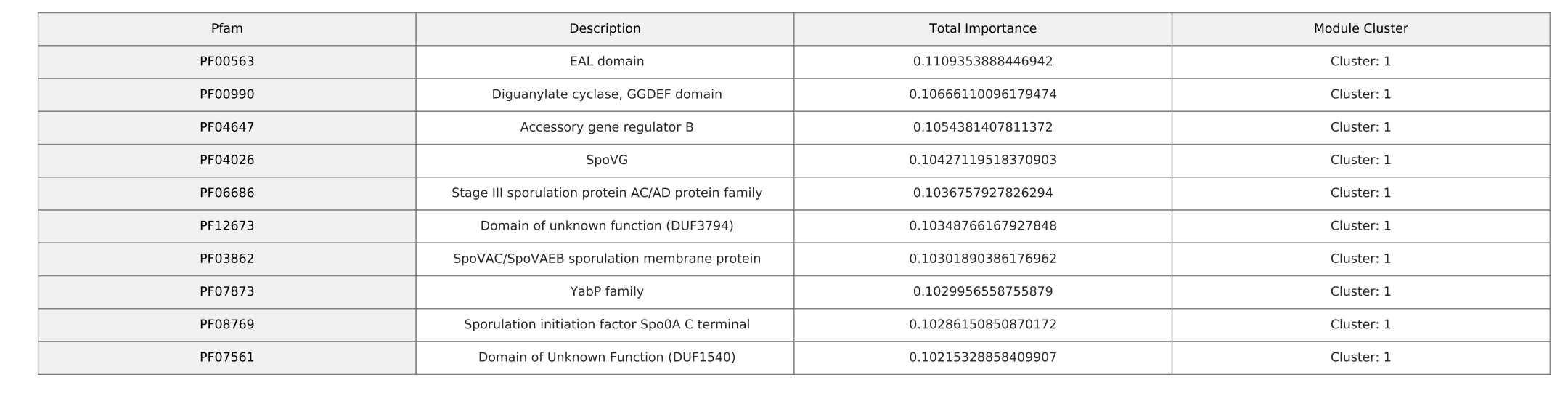
**

**
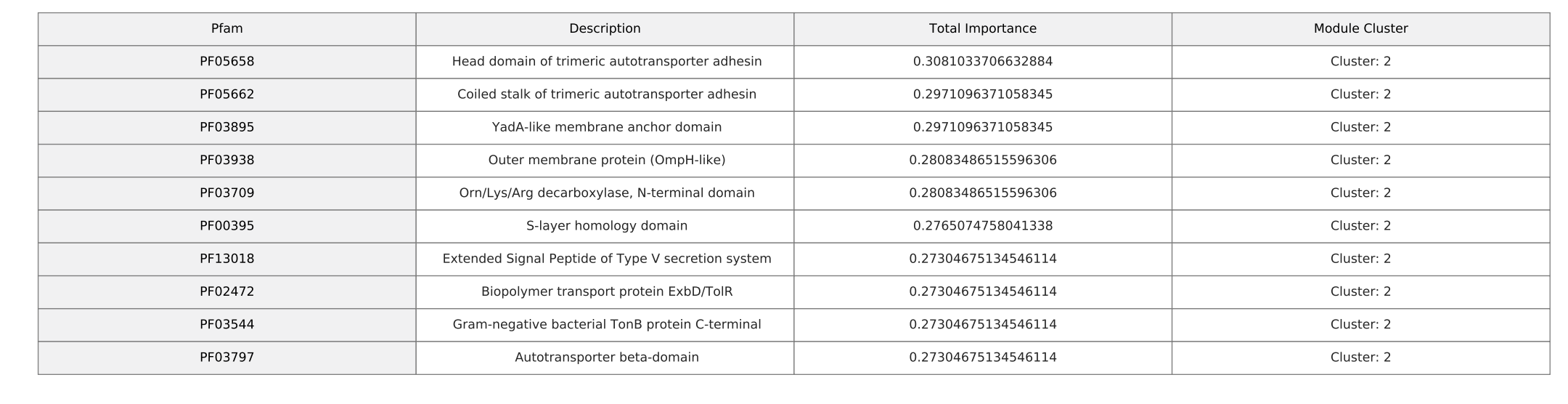
**

**
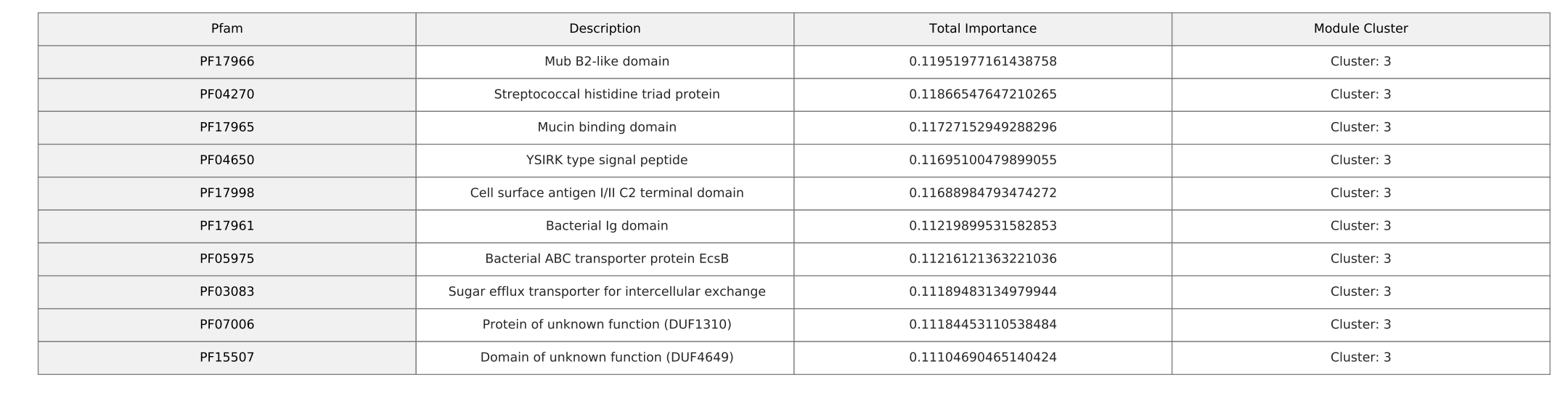
**

**
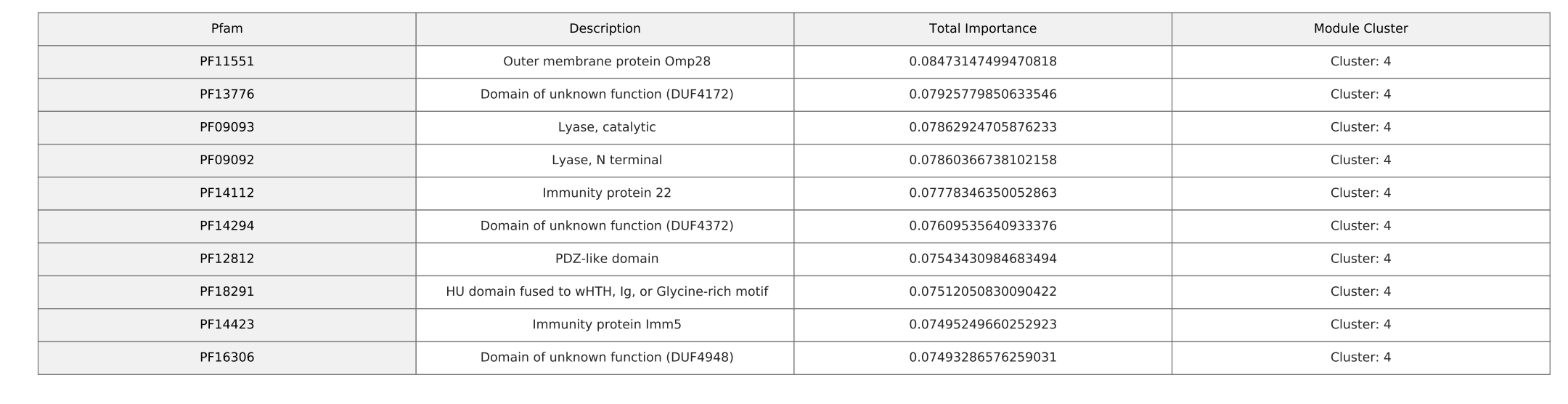
**

**
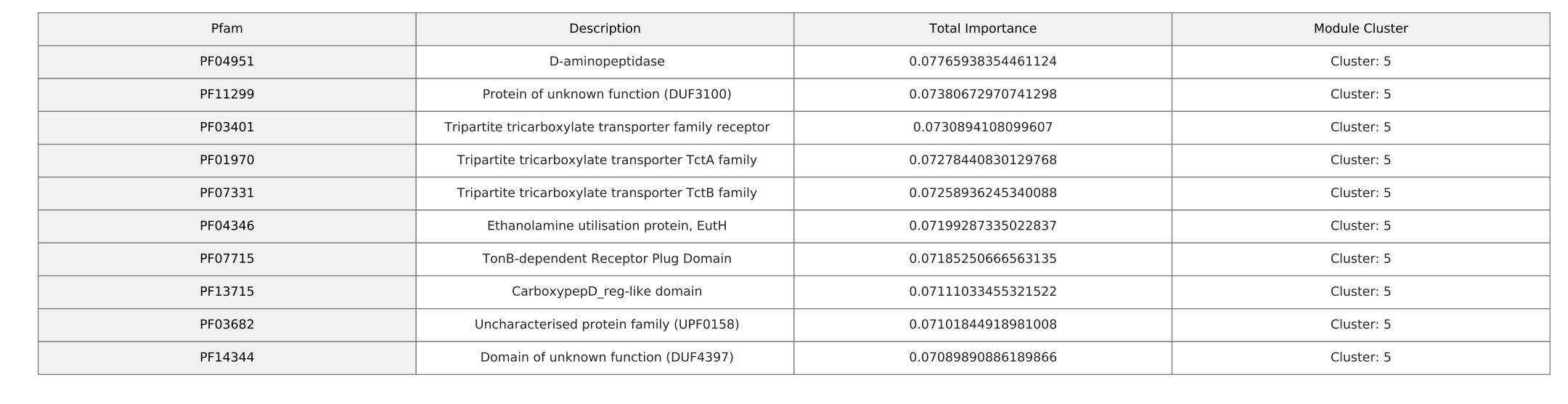
**

**b**

**
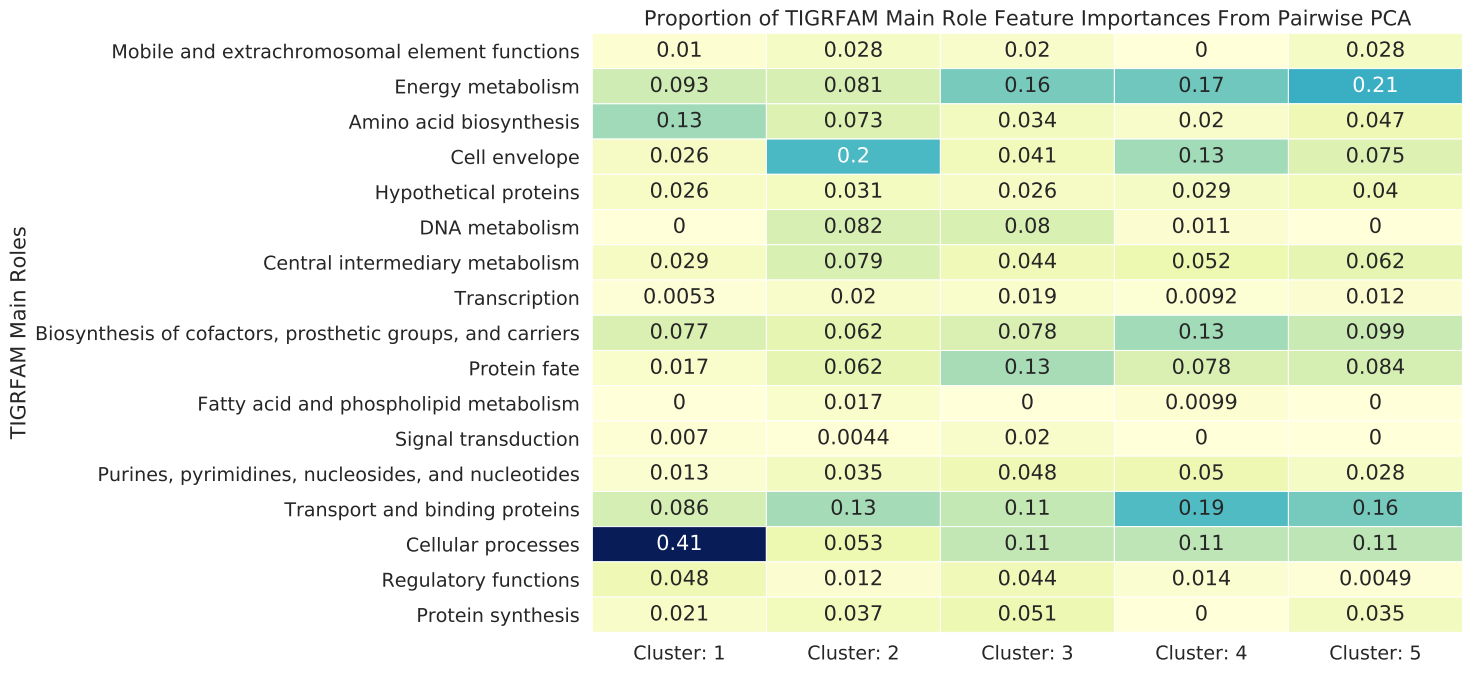
**

**c**

**
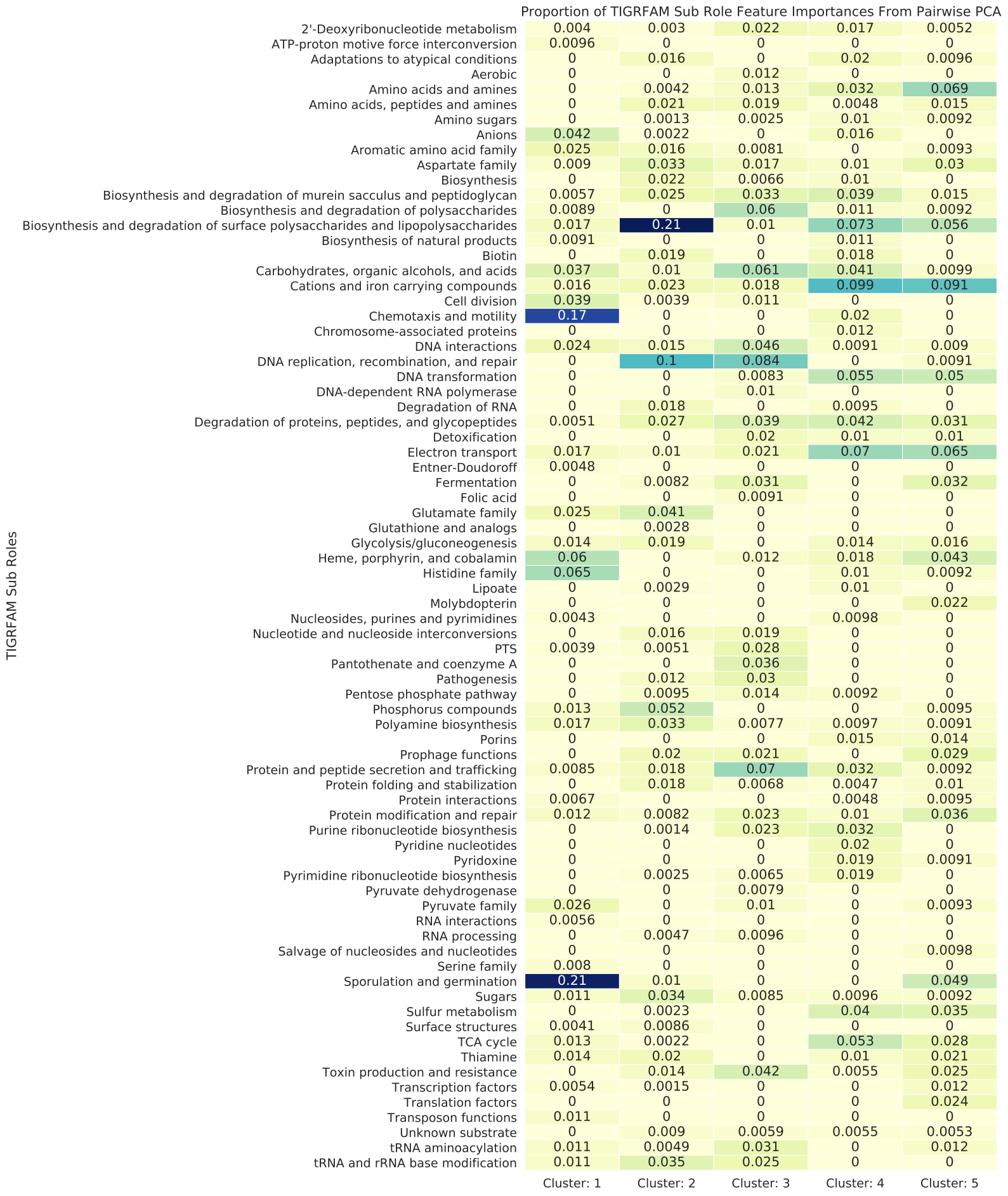
**

**Supplemental 8: Module Cluster Functional Profiling**

**a.** Table showing the top 10 Pfams of each cluster with the highest total importance. Pfam total importance was produced from pairwise PCA of module cluster functional profiles (see methods). **b.** Heatmap showing the proportion of the total top 100 TIGRFAM feature importances TIGRFAM main roles account for within clusters. **c.** Heatmap showing the proportion of the total top 100 TIGRFAM feature importances TIGRFAM sub roles account for within clusters.

**
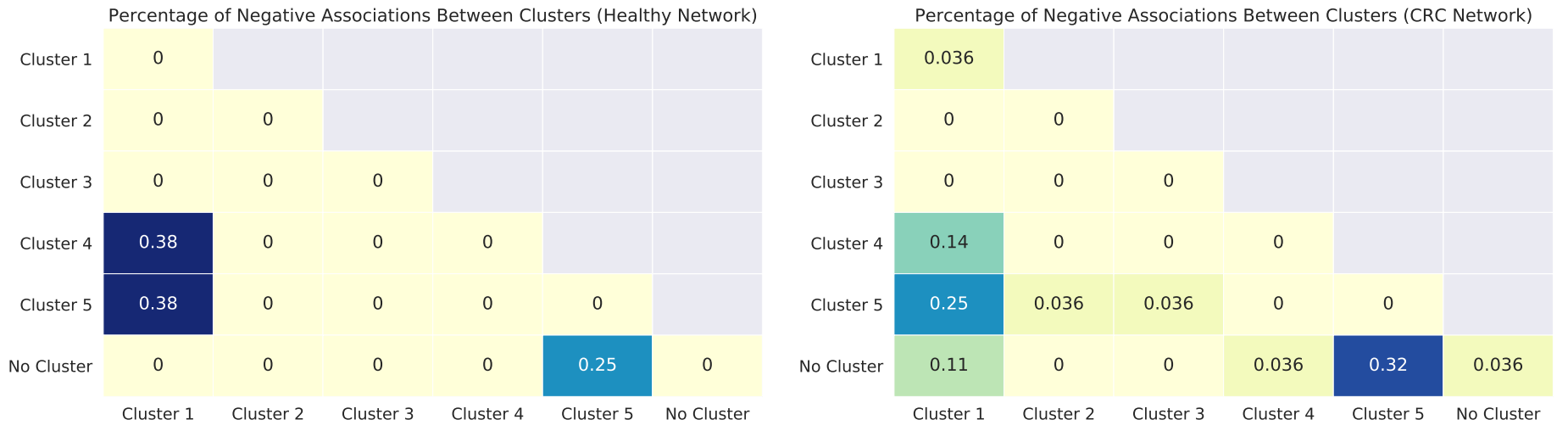
**

**Supplemental 9: Negative Associations Between Network Module Clusters**

Heatmaps showing the proportion of negative associations occurring between species from each module cluster type within Healthy and CRC networks. Within the CRC network we see the first occurrence of an intra-cluster negative association between species (cluster 1).

**
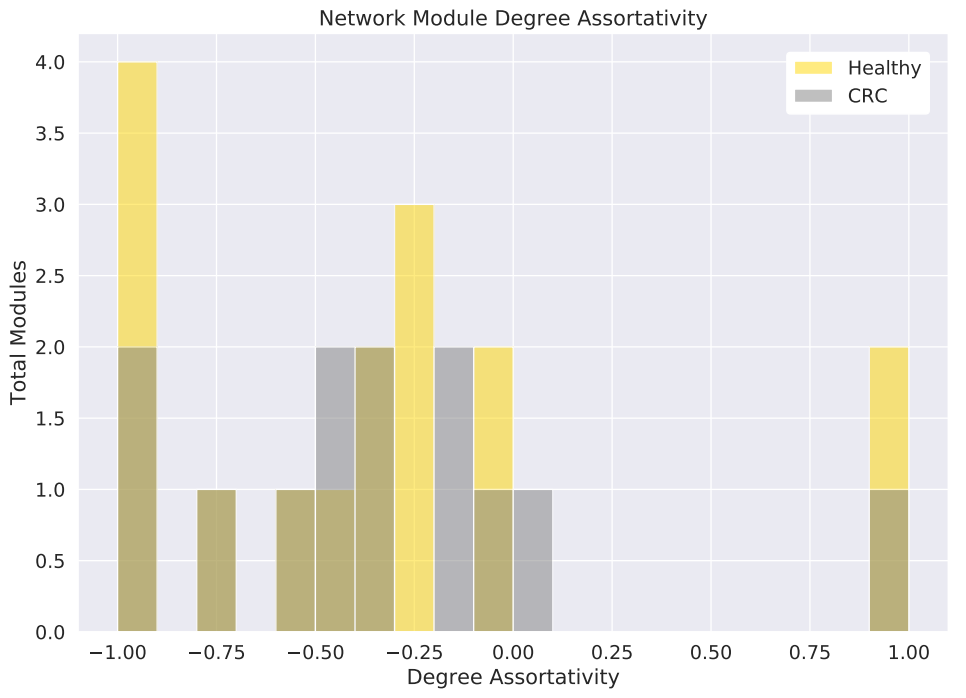
**

**Supplemental 10: Degree Assortativity of Network Modules**

Distribution of the degree assortativity of module sub graphs within Healthy and CRC networks. This graph shows most modules within networks have a degree assortativity below 0 suggesting ‘Hub’ nodes exist within these modules.


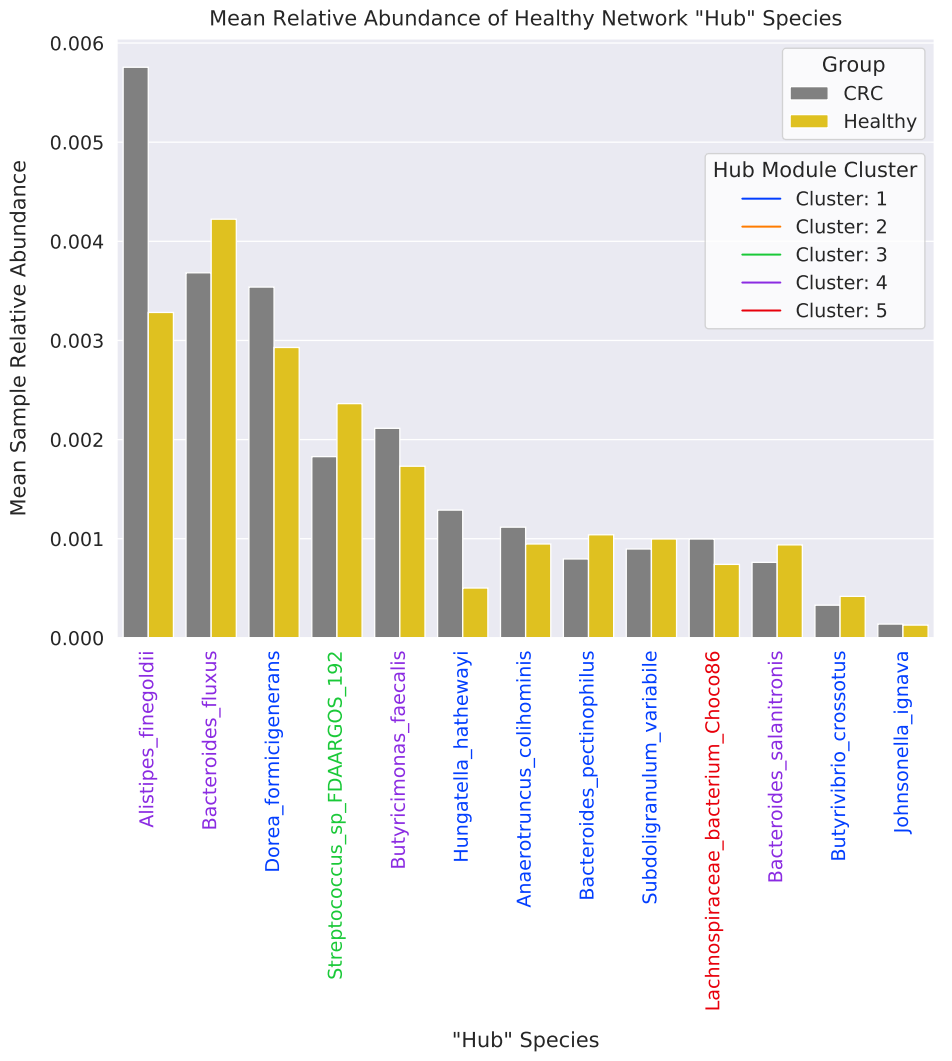
**a**

**
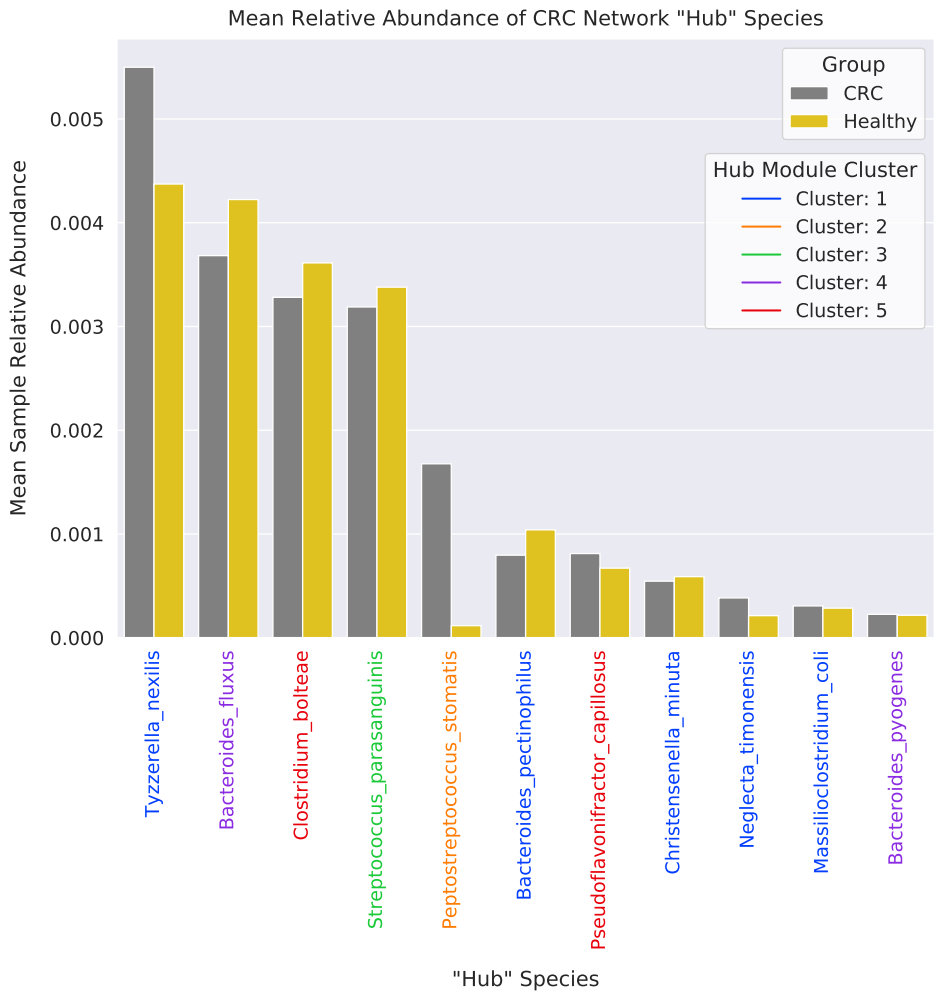
**

**b**

**Supplemental 11: Mean Relative Abundance of “Hub” species within Healthy and CRC Networks.**

The mean relative abundance of “Hub” nodes found within Healthy (**a**) and CRC (**b**) Networks. The color of the “Hub” species name represents which cluster their module was associated with (Cluster 1-5). Only within the CRC network were oral microbes found to be “Hubs”.

**
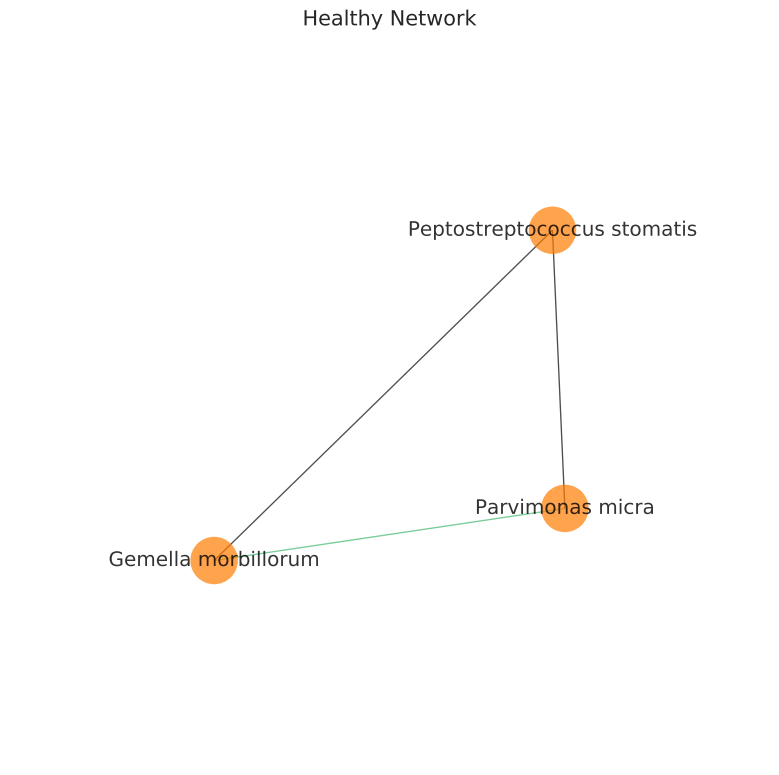

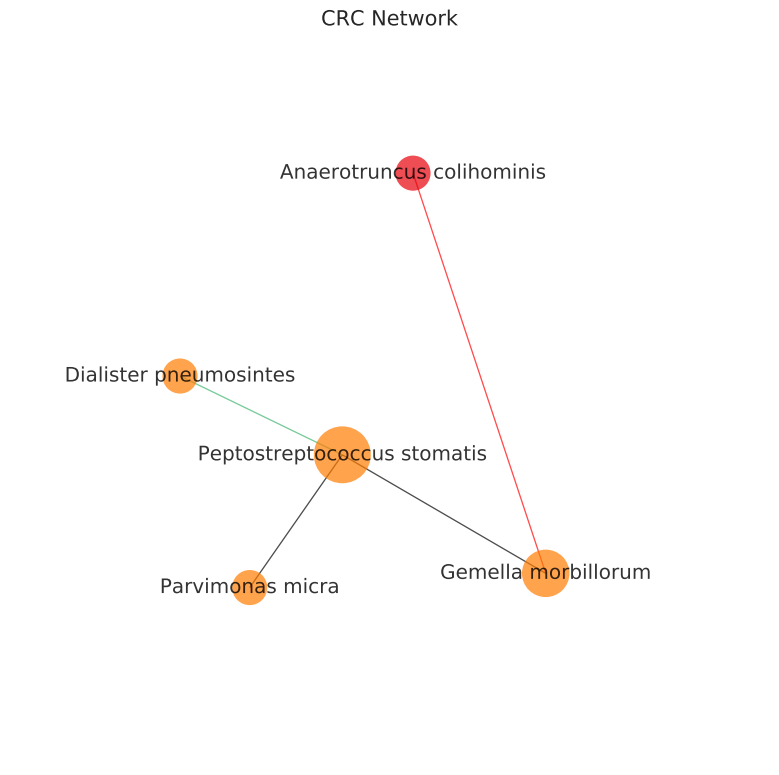
**

**Supplemental 12: The ‘Pathobiont’ Oral Microbe Module Within Healthy and CRC Networks**

Modules of ‘pathobiont’ oral microbes within the Healthy and CRC Networks. The CRC oral microbe module shows the addition of *Dialister* *pneumosintes* and a change in topology. Within this network *Peptostreptococcus* *stomatis* has become a ‘Hub’ node. This module has also gained a negative association with *Anaerotruncus* *colihominis* a ‘Hub’ only within the Healthy network. Node color denotes the module cluster this species is found within (Orange = Cluster 2, Red = Cluster 5). Node are sized by their degree (total associations). Black edges are positive associations found within both networks, while green and red are positive or negative associations unique to networks, respectively.

**a.**

**
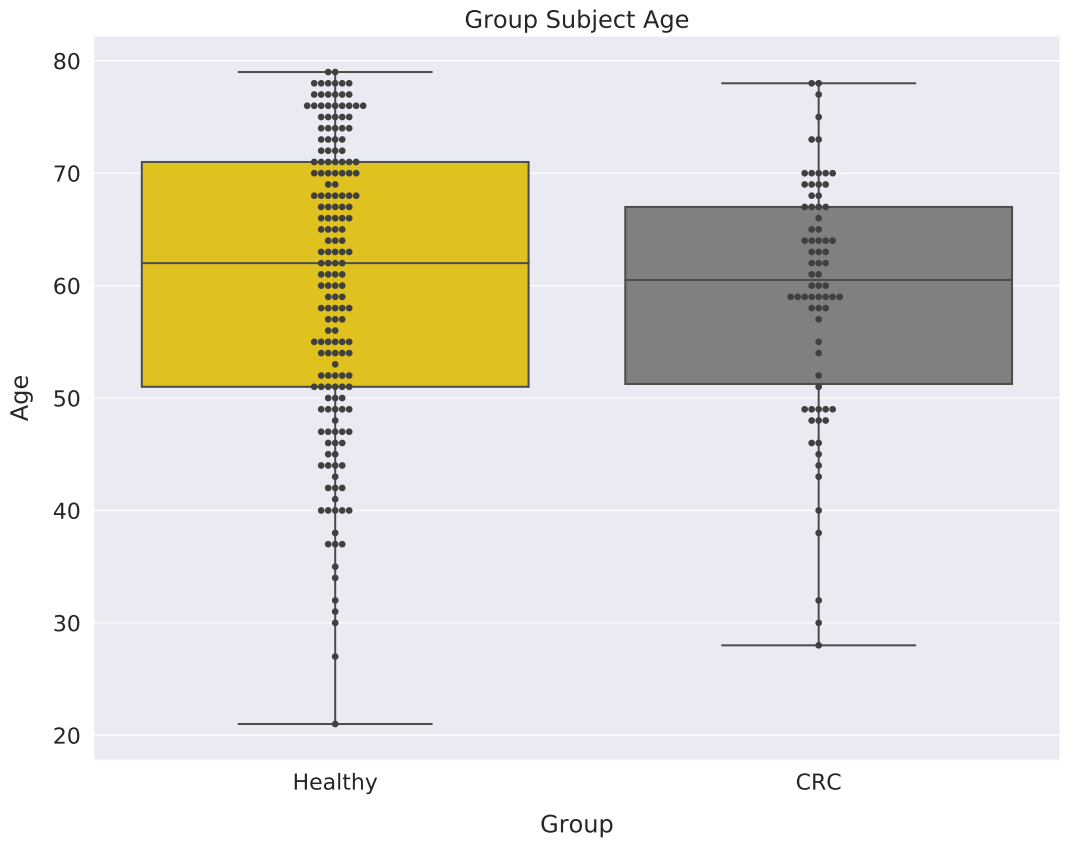
**


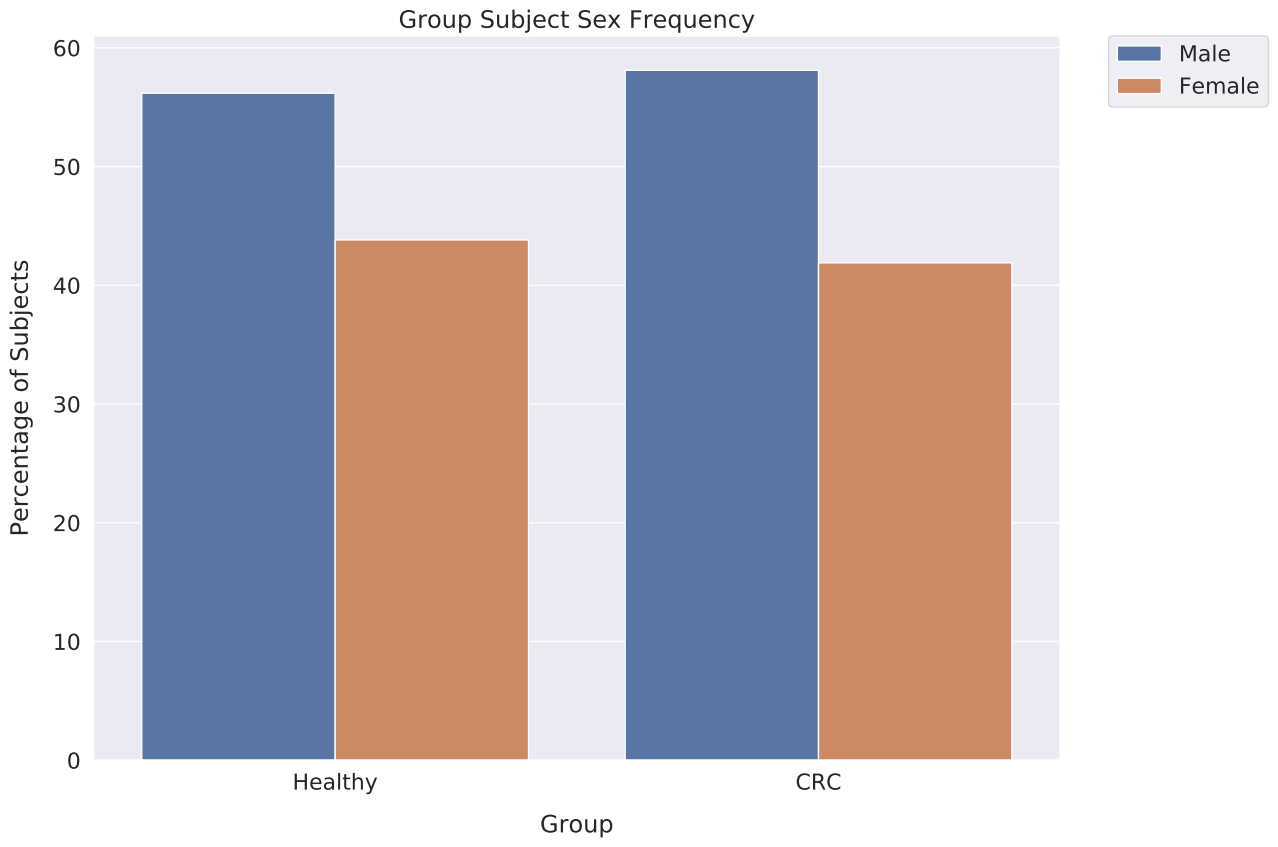
**b.**

**Supplemental 13: Subject Age and Sex Distributions within Groups.**

**a.** Boxplots of Healthy and CRC sample group subject age. **b.** Barplots of Healthy and CRC sample group subject sex frequencies. Both the age (Healthy sample group median age: 62; CRC sample group median age: 61) and sex (Healthy: Male 56.18%, Female: 43.82 %; CRC: Male: 58.11 %, Female: 41.89) of subjects are similar in both sample groups.
